# Weakly nucleophilic potassium aryltrifluoroborates in palladium-catalyzed Suzuki–Miyaura reactions: relative reactivity of K[4-RC_6_F_4_BF_3_] and the role of silver-assistance in acceleration of transmetallation

**DOI:** 10.3762/bjoc.11.68

**Published:** 2015-05-04

**Authors:** Vadim V Bardin, Anton Yu Shabalin, Nicolay Yu Adonin

**Affiliations:** 1 N.N. Vorozhtsov Novosibirsk Institute of Organic Chemistry, SB RAS, Acad. Lavrentjev Ave. 9, Novosibirsk, 630090, Russian Federation; 2 Novosibirsk State University, Pirogova str., 2, Novosibirsk, 630090, Russian Federation; 3 G.K. Boreskov Institute of Catalysis, SB RAS, Acad. Lavrentjev Ave. 5, Novosibirsk, 630090, Russian Federation

**Keywords:** potassium polyfluoroaryltrifluoroborate, silver(I) acceleration, Suzuki–Miyaura reaction

## Abstract

Small differences in the reactivity of weakly nucleophilic potassium aryltrifluoroborates are revealed in the silver-assisted Pd-catalyzed cross-coupling of K[4-RC_6_F_4_BF_3_] (R = H, Bu, MeO, EtO, PrO, iPrO, BuO, *t*-BuO, CH_2_=CHCH_2_O, PhCH_2_O, PhCH_2_CH_2_O, PhO, F, pyrazol-1-yl, pyrrol-1-yl, and indol-1-yl) with ArX (4-BrC_6_H_4_CH_3_, 4-IC_6_H_4_F and 3-IC_6_H_4_F). An assumed role of silver(I) compounds Ag*_m_*Y (Y = O, NO_3_, SO_4_, BF_4_, F) consists in polarization of the Pd–X bond in neutral complex ArPdL*_n_*X with the generation of the related transition state or formation of [ArPdL*_n_*][XAg*_m_*Y] with a highly electrophilic cation and subsequent transmetallation with the weakly nucleophilic borate. Efficiency of Ag*_m_*Y as a polarizing agent decreases in order Ag_2_O > AgNO_3_ ≈ Ag_2_SO_4_ > Ag[BF_4_] > AgF. No clear correlation between the reactivity of K[4-RC_6_F_4_BF_3_] and substituent electron parameters, σ_I_ and σ_R_°, of the aryl group 4-RC_6_F_4_ was found.

## Introduction

The palladium-catalyzed reaction of organoboron compounds with C-electrophiles (Suzuki–Miyaura reaction) is one of the most intensively studied processes of the carbon–carbon bond formation. Organoboronic acids, their esters and organotrifluoroborates are partners in these reactions and the choice of the desired reagent depends on the specific requirements in each particular case [1–3]. Organoboron reagents containing an electron-poor organic moiety exhibit a low reactivity under the usual cross-coupling conditions [3–9] and the target products are formed in low yield and/or are contaminated with byproducts.

**Scheme 1:**
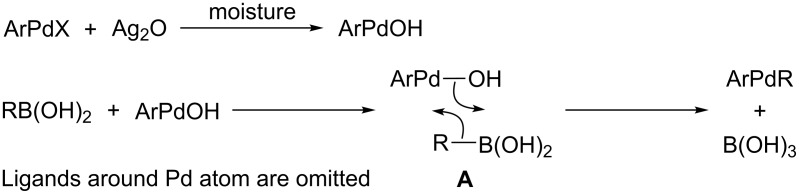
The assumed silver(I) oxide assisted transmetallation with organoboronic acids.

Reactions of weakly nucleophilic organoboron reagents (alkyl- and cyclopropylboronic acids and esters [10–14], alken-1-ylboronic acids and esters [15–16], some arylboronic acids [17–19], K[CF_2_=CFBF_3_] [19]) often are accelerated by the addition of stoichiometric amounts of Ag_2_O. Initially this phenomenon was reported by Kishi et al. [16] for the cross-coupling of alkenylboronic acids with alkenyl iodides in the presence of Ag_2_O and elucidated by formation of AgOH which acts like aqueous KOH or TlOH. Korenaga et al. [20] have studied Pd-catalyzed cross-coupling of C_6_F_5_B(OH)_2_ with aryl halides in the presence of Ag_2_O and assumed the generation of a hydroxy–palladium complex with higher ability to transmetallation under the action of the corresponding organoboron reagent than ArPdL_2_X. For example, complex *trans*-C_6_F_5_Pd(PEt_3_)_2_OH is formed in the reaction of *trans*-C_6_F_5_Pd(PEt_3_)_2_I with Ag_2_O in toluene–water and undergoes transmetallation with 4-MeOC_6_H_4_B(OH)_2_ [21]. The subsequent reductive elimination leads to the corresponding polyfluorobiphenyl. In contrary, the reaction of *trans*-C_6_F_5_Pd(PEt_3_)_2_I with 2,4,6-C_6_F_3_H_2_B(OH)_2_ and Ag_2_O leads to an unsymmetrical diarylpalladium complex *trans*-(C_6_F_5_)Pd(PEt_3_)_2_(2,4,6-C_6_F_3_H_2_) in 92% yield. The latter is thermally stable and does not produce the cross-coupling product even upon heating in toluene at 100 °C for 24 h [21]. The authors suggested that acceleration of these reactions by silver(I) oxide results in the generation of a hydroxy–palladium complex with a higher ability to transmetallation, to coordinate to the organoboronic acid with the three-coordinated boron atom to ArPdL_2_OH and subsequent transmetallation (Scheme 1).

However, some experimental facts indicate the multilateral role of silver(I) compounds Ag*_m_*Y in the acceleration of the Suzuki–Miyaura cross-coupling. Beside generation of ArPdL*_n_*OH, Ag*_m_*Y may act as a Lewis acid producing complex [RPdL*_n_*][XAg*_m_*Y] with a highly electrophilic cation. The reactions often are performed in polar aprotic solvents (MeCN, THF, acetone) and the complexes exist in solutions as solvates [RPdL*_n_*(solv.)][XAg*_m_*Y]. Some complexes are much more stable and can be isolated [22–23], although for preparative aims they usually are generated in situ. This approach has been successfully used for arylation of alkenes, alkynes, insertion of CO species [23–31], polyfluoroarenes and thiophenes [32].

The effect of the counterion Y in silver(I) compounds Ag*_m_*Y on the rate of the cross-coupling was slightly investigated. For example, the rates of the CO insertion into cationic methylpalladium complex [MePd(PMe_3_)_2_][XAg*_m_*Y] generated from MePd(PMe_3_)_2_Cl, Ag[BF_4_] or Ag[PF_6_] in acetone are equal to 23 ∙ 10^−5^ and 24 ∙ 10^−5^ s^−1^. The use of AgOTf (11 ∙ 10^−5^ s^−1^) or AgNO_3_ (5.9 ∙ 10^−5^ s^–1^) causes a decrease in the reactivity towards the CO insertion [28,33]. The similar effect of anions Y = BF_4_, OTf, PF_6_ was observed in the formation of complexes [RPd(PMe_3_)_2_(solv.)][XAg*_m_*Y] and [RPd(PMe_3_)(solv.)_2_][XAg*_m_*Y] [34].

In continuation of systematic research of the Suzuki–Miyaura cross-coupling reaction of weakly nucleophilic organotrifluoroborates we report here the study of the relative reactivity of K[4-RC_6_F_4_BF_3_] in the Pd-catalyzed reactions with some aryl bromides and iodides in the presence of Ag_2_O. The borates were chosen as model organoboron reagents because of tuning electronic properties of 4-RC_6_F_4_ groups with varied R = H, Bu, MeO, EtO, PrO, iPrO, BuO, *t*-BuO, CH_2_=CHCH_2_O, PhCH_2_O, Ph(CH_2_)_2_O, PhO, pyrazol-1-yl (Prz), pyrrol-1-yl (Pyr), indol-1-yl (Ind), imidazol-1-yl (Im), and benzamidazol-1-yl (Bim) and the equal steric requirements near reaction site C^1^–BF_3_. The relative reactivity was estimated (a) from the yield of biphenyl 4-RC_6_F_4_–Ar vs C_6_F_5_–Ar and (b) from the rate of consumption of K[4-RC_6_F_4_BF_3_] and compared with the substituent electron parameters (SEP) of the 4-RC_6_F_4_ group. The efficiency of some other silver(I) compounds (AgNO_3_, Ag_2_SO_4_, Ag[BF_4_], and AgF) in the cross-coupling was examined too.

## Results

**Scheme 2:**
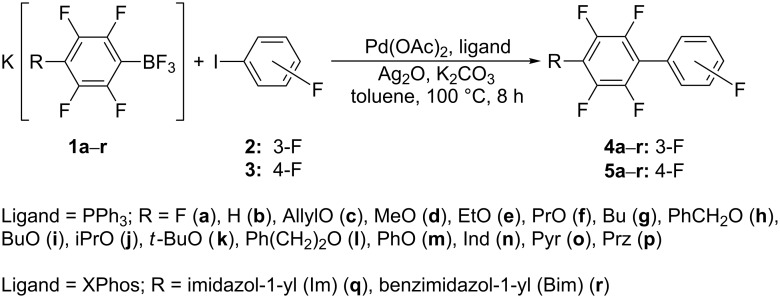
Cross-coupling of K[4-RC_6_F_4_BF_3_] (**1a–r**) with 3-IC_6_H_4_F (**2**) and 4-IC_6_H_4_F (**3**).

All reactions were carried out under the previously elaborated optimal conditions [35]. The cross-coupling of K[4-RC_6_F_4_BF_3_] (**1a–r**) with 1-fluoro-3-iodobenzene (**2**) produces pentafluorobiphenyls 4-(3'-FC_6_H_4_)C_6_F_4_R in 80–99% yield (Scheme 2 and Table 1). Pentafluorobiphenyls 4-(4'-FC_6_H_4_)C_6_F_4_R were obtained from K[4-RC_6_F_4_BF_3_] and 1-fluoro-4-iodobenzene (**3**). Notably borates K[4-RC_6_F_4_BF_3_] with R = H (**1b**), AllylO (**1c**) and azolides (**1n–r**) (see Table 1, entries 2, 3, 14–18) give much lower yields of the corresponding biphenyls than K[C_6_F_5_BF_3_] (**1a**) (Table 1, entry 1) [19].

**Table 1:** Preparation of biphenyls **4a–r** and **5a–r**.

Entry	K[4-RC_6_F_4_BF_3_]	Isolated yield (%)	
		**4**	**5**

1	**1a** (R = F)	99	99
2	**1b** (R = H)	26	24
3	**1c** (R = AllylO)	34	18
4	**1d** (R = MeO)	90	94
5	**1e** (R = EtO)	98	97
6	**1f** (R = PrO)	82	96
7	**1g** (R = Bu)	89	50
8	**1h** (R = PhCH_2_O)	86	73
9	**1i** (R = BuO)	87	99
10	**1j** (R = iPrO)	82	97
11	**1k** (R = *t*-BuO)	98	99
12	**1l** (R = Ph(CH_2_)_2_O)	79	71
13	**1m** (R = PhO)	98	96
14	**1n** (R = Ind)	91	42
15	**1o** (R = Pyr)	84	20
16	**1p** (R = Prz)	47	35^a^
17	**1q** (R = Im)	10^b, c^	12^b, d^
18	**1r** (R = Bim)	20^b, e^	20^b, f^

^a^Byproduct 2,3,5,6-C_6_F_4_HPrz (3%); ^b^phosphine XPhos was used instead of PPh_3_; ^c^byproduct 2,3,5,6-C_6_F_4_HIm (7%); ^d^byproduct 2,3,5,6-C_6_F_4_HIm (7%); ^e^byproduct 2,3,5,6-C_6_F_4_HBim (5%); ^f^byproduct 2,3,5,6-C_6_F_4_HBim (14%).

**Scheme 3:**
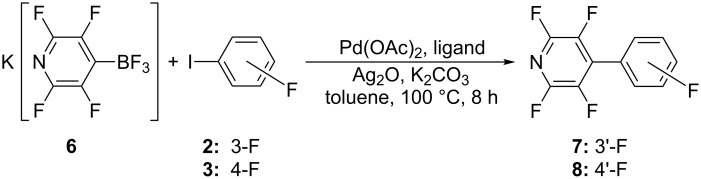
Attempted synthesis of **7** (3’-F) and **8** (4’-F) by cross-coupling reaction.

Potassium 2,3,5,6-tetrafluoropyridyltrifluoroborate (**6**) exhibits extremely low reactivity toward both **2** and **3** gives the corresponding 4-(3'-fluorophenyl)- (**7**) and 4-(4'-fluorophenyl)- (**8**) -2,3,5,6-tetrafluoropyridines with yields no more than 5% (^19^F NMR) (Scheme 3). It should be noted that 2,3,5,6-tetrafluoropyridine was not observed in the reaction mixtures in both cases.

**Scheme 4:**
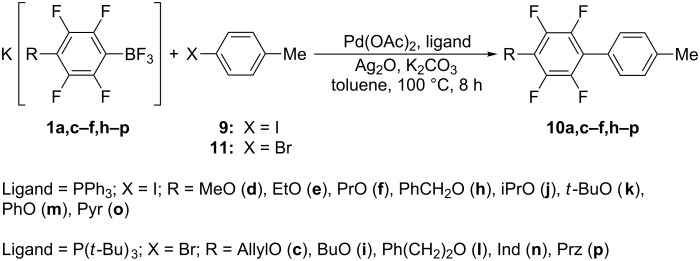
Synthesis of biphenyls **10a,c–f,h–p**.

The use of the electron-rich C-electrophile, 4-IC_6_H_4_CH_3_ (**9**), instead of **3** leads to biphenyls 4-(4'-CH_3_C_6_H_4_)C_6_F_4_R (**10c–f,h–p**) in 60–80% preparative yields (see Table 2, entries 2–14) while 4-CH_3_C_6_H_4_C_6_F_5_ (**10a**) was isolated in 93% yield (see Table 2, entry 1) [19]. Although aryl bromides are less reactive than iodides, the substitution of 4-IC_6_H_4_CH_3_ (**9**) for 4-BrC_6_H_4_CH_3_ (**11**) does not affect the yields of the corresponding biphenyls (Scheme 4).

**Table 2:** Preparation of biphenyls **10a,c–f,h–p**.

Entry	K[4-RC_6_F_4_BF_3_]	Isolated yield of **10** (%)	Entry	K[4-RC_6_F_4_BF_3_]	Isolated yield of **10** (%)

1	**1a** (R = F)	93	8	**1j** (R = iPrO)	71
2	**1c** (R = AllylO)	25^a^	9	**1k** (R = *t*-BuO)	62
3	**1d** (R = MeO)	80	10	**1l** (R = Ph(CH_2_)_2_O)	93
4	**1e** (R = EtO)	70	11	**1m** (R = PhO)	65
5	**1f** (R = PrO)	83	12	**1n** (R = Ind)	91
6	**1h** (R = PhCH_2_O)	43^b^	13	**1o** (R = Pyr)	73
7	**1i** (R = BuO)	97	14	**1p** (R = Prz)	99

^a^Byproduct 2,3,5,6-C_6_F_4_HOCH_2_CH=CH_2_ (18%); ^b^byproduct 2,3,5,6-C_6_F_4_HOCH_2_Ph (28%).

**Scheme 5:**
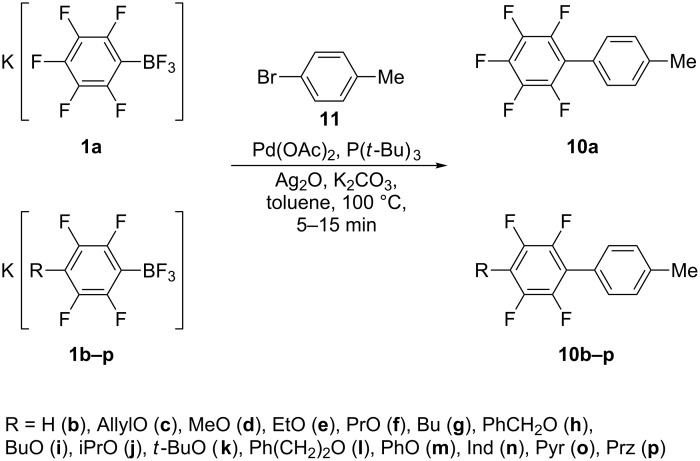
Pd-catalyzed cross-coupling of **1a** and salts **1b–p** (1:1) with **11** (the results are presented in Table 3 and outlined in the Discussion section).

The obtained results show a similar or slightly reduced reactivity of the majority of K[4-RC_6_F_4_BF_3_] (R ≠ F) with respect to salt **1a**. Exceptions are borates with R = H (**1b**), CH_2_=CHCH_2_O (**1c**) and azolides (**1n–r**), which produce cross-coupling products in low to moderate yields because of the side reactions. Highly tolerant borate **6** also gives the product in a low yield, but its conversion is low too. Hence, the data based on an isolated yield of biphenyls **4a–r**, **5a–r**, or **10a**,**c–f,h–p** are not a convenient measure for the quantitative estimation of the relative reactivity. We hoped to get more accurate data by the concurrent cross-coupling of equimolar mixtures of K[C_6_F_5_BF_3_] (**1a**) and K[4-RC_6_F_4_BF_3_] (**1b–p**) with **11**. The reactions were carried out over a short period (5–15 min) and the product ratios were determined by analyzing the crude reaction mixtures with ^19^F NMR spectroscopy. The relative reactivity was determined as C^rel^ = C^R^ / C^F^ where C^R^ and C^F^ are the yield (in mmol) of 4-(4'-CH_3_C_6_H_4_)C_6_F_4_R from salts **1b–p** and 4'-CH_3_C_6_H_4_C_6_F_5_ from **1a**, respectively (Scheme 5, Table 3).

**Table 3:** Relative reactivity C^rel^ of K[4-RC_6_F_4_BF_3_] in the cross coupling with **11**.^a,b^

Entry	K[4-RC_6_F_4_BF_3_]	C^R^	C^F^	C^rel^

1	**1c** (R = AllylO)	13	22	0.59
2	**1g** (R = Bu)	27	40	0.68
3	**1f** (R = PrO)	22	32	0.68
4	**1d** (R = MeO)	22	29	0.76
5	**1h** (R = PhCH_2_O)	25	32	0.78
6	**1b** (R = H)	22	28	0.79
7	**1i** (R = BuO)	23	28	0.82
8	**1k** (R = *t*-BuO)	26	29	0.90
9	**1j** (R = iPrO)	23	25	0.92
10	**1e** (R = EtO)	31	33	0.94
11	**1m** (R = PhO)	20	21	0.95
12	**1n** (R = Ind)	26	27	0.96
13	**1l** (R = Ph(CH_2_)_2_O)	22	22	1.00
14	**1o** (R = Pyr)	26	25	1.04
15	**1p** (R = Prz)	26	25	1.04

^a^Conditions: Pd(OAc)_2_, P(*t*-Bu)_3_, Ag_2_O, K_2_CO_3_ in toluene, 100 °C, 5–15 min; ^b^C^rel^ = C^R^/C^F^, where C^R^ = 4-(4'-CH_3_C_6_H_4_)C_6_F_4_R (mmol)/K[4-RC_6_F_4_BF_3_] (0.20 mmol) and C^F^ = 4'-CH_3_C_6_H_4_C_6_F_5_ (mmol)/K[C_6_F_5_BF_3_] (0.20 mmol).

**Scheme 6:**
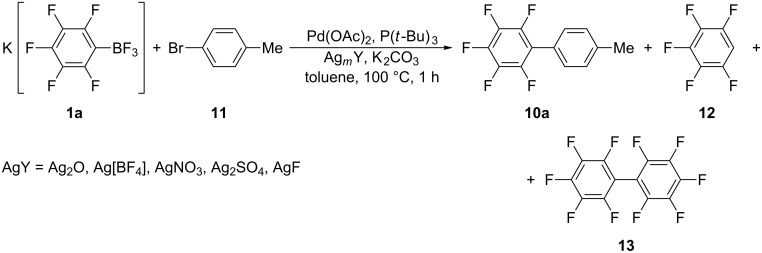
The cross-coupling of **1a** with **11** in the presence of different silver(I) compounds.

The above experiments were performed using silver(I) oxide. For better understanding the role of Ag^+^ we estimated the relative efficiency of some other silver(I) compounds under identical conditions (Scheme 6) (Table 4).

**Table 4:** Cross-coupling of **1a** with **11** in the presence of different silver(I) compounds.

Entry	Conversion of **1a** ( %)	Ag*_m_*Y	Yield (%)^a,b^		
			**10a**	**12**	**13**

1	69	Ag_2_O	99	1	0
2	79	AgNO_3_	20	27	27
3	75	Ag_2_SO_4_	20	28	26
4	40	Ag[BF_4_]	40	60	0
5	19	AgF	51	43	3

^a^Yields were determined by ^19^F NMR; ^b^calculated on reacted **1a**.

The presented data demonstrate clearly that AgNO_3_, Ag_2_SO_4_, Ag[BF_4_], and AgF salts are less appropriate promotors for the palladium catalyzed cross-coupling than Ag_2_O. The significant contribution of the side reactions (hydrodeboration and homo-coupling) results in decreasing yields of **10a** and hinders isolation of the desired product (see Table 4).

## Discussion

**Scheme 7:**
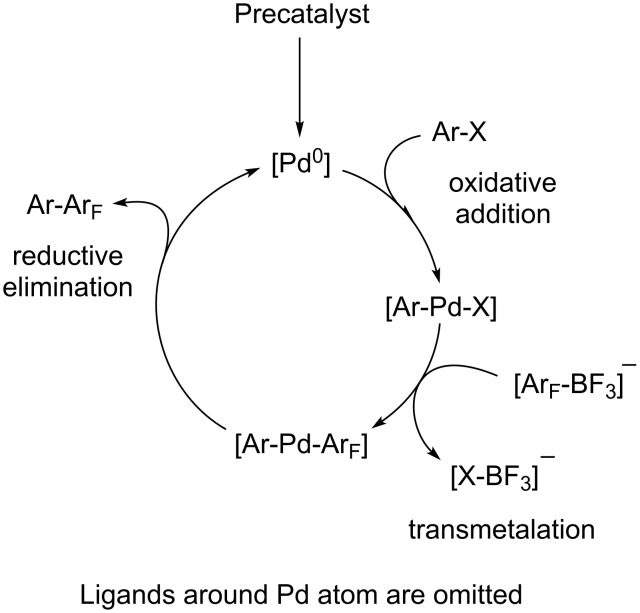
General concept of Pd-catalyzed Suzuki–Miyaura reaction.

The general concept of the Pd-catalyzed Suzuki–Miyaura (SM) reaction applied to the cross-coupling of K[4-RC_6_F_4_BF_3_] with ArX is presented in Scheme 7.

The first step is the generation of neutral complex ArPdX by the oxidative addition of ArX to Pd(0) species. This step precedes the subsequent transformation of ArPdX and does not influence the reactivity of organoboron partner K[4-RC_6_F_4_BF_3_] as well as its behavior in transmetallation and/or reductive elimination steps. For the further consideration we need to clarify the nature of the active organoboron partner. Despite of many publications [2,6–7,36–37] in this field it is not yet fully understood. For illustration, we refer to two examples. Molander argues for direct transmetallation of ArPdBr by aryltrifluoroborates in aprotic anhydrous THF in the presence of trialkylamine as a base or in alcohols [4]. Alternatively, the Pd-catalyzed cross-coupling of K[ArBF_3_] in aqueous THF is proved to proceed through stepwise hydrolysis to produce more reactive [ArBF*_n_*(OH)_3-_*_n_*]^−^ or ArB(OH)_2_ [35,38]. However, special experiments showed that K[C_6_F_5_BF_3_] retards toward K_2_CO_3_ [39] as well as K_2_CO_3_ in a mixture with catalytic amounts of Pd(OAc)_2_ in refluxing MeOH [19]. Resistance of K[C_6_F_5_BF_3_] (**1a**) towards K_2_CO_3_ and Ag_2_O in toluene (without palladium catalyst and phosphine ligand) is confirmed by stirring the corresponding suspensions at 100 °C for 10 min. These facts make it possible to reject any transformation of the [BF_3_]^−^ moiety before the transmetallation of the palladium-containing intermediate generated in the previous step of the catalytic cycle by K[4-RC_6_F_4_BF_3_]. Potassium carbonate scavenges toluene-soluble acidic impurities formed in the reaction and does not participate in the other steps [19,35].

The boron atom in K[4-RC_6_F_4_BF_3_] is coordinately saturated and transmetallation via transition state **A** (Scheme 1) is impossible. An alternative pathway is the polarization of the palladium–X bond by Ag^+^ and the generation of complex [ArPdL*_n_*][XAg*_m_*Y] with a highly electrophilic cation which is attacked by carbon atom C-1 of K[C_6_F_5_BF_3_] (**1a**). In toluene salt **1a** is consumed within 3 h to give 2,3,4,4’,5,6-hexafluorobiphenyl (**5a**) in 70–92% yield [19], while in the polar coordinating solvents (DME, DMF) the Ag_2_O-assisted cross-coupling of **1a** with **3** leads to formation of **5a** at unsatisfactory conversions (22–38%) and low yields (10–22%). This indicates that the strong solvation of the Pd atom with DME or DMF reduces electrophilicity of [ArPdL*_n_*][X] as compared with the electrophilicity in the case of weakly coordinating toluene.

**Scheme 8:**
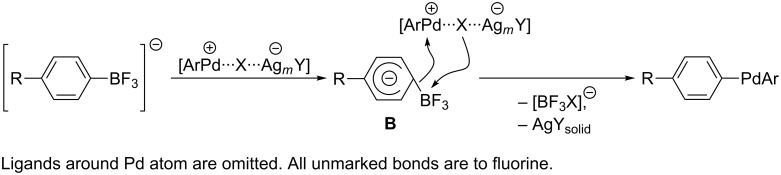
Assumed silver(I)-assisted transmetallation of weakly nucleophilic aryitrifluoroborates.

K[C_6_F_5_BF_3_] (**1a**) and Ag*_m_*Y are insoluble in toluene and, very likely, the transmetallation proceeds on the surface of the silver(I) compounds. Both the aryltrifluoroborate anion and ArPdL*_n_*X can be adsorbed on the surface of solid Ag*_m_*Y due to the interaction with Ag atoms (acidic Lewis centers). This phenomenon causes an increase in the electrophilicity of the Pd atom and bond rearrangement through transition state **B** to give the diarylpalladium species (Scheme 8). Perhaps, transformations presented in Scheme 8 are parallel, i.e., the transmetallation proceeds simultaneously with polarization of ArPdL*_n_*X.

There is a popular notion that the transmetallation gives both *cis*- and *trans*-ArPdL*_n_*Ar'. First of them is low stable and undergoes easy reductive elimination to biphenyl. *trans*-ArPdL*_n_*Ar' can react only after transformation to the corresponding *cis*-isomer [40–41]. Complexes with polyfluorinated aryl groups are highly stable and do not isomerize to the *cis*-isomer as well as do not form the cross-coupling products. Thus, independently prepared complexes *trans*-C_6_F_5_PdL_2_(2,4,6-C_6_F_3_H_2_) (L = PEt_3_, PMe_2_Ph, PMePh_2_) do not undergo any changes even after heating in toluene at 100 °C for 24 h [21]. These facts lead to assume either instability of *trans*-(4-RC_6_F_4_)PdL_2_Ar towards isomerisation to *cis*-isomer or the direct formation of reactive complex *cis*-(4-RC_6_F_4_)PdL_2_Ar during the transmetallation with closely related borates K[4-RC_6_F_4_BF_3_] (Scheme 8).

At the next step, *cis*-(4-RC_6_F_4_)PdL_2_Ar undergoes reductive elimination which includes the carbon–palladium bond cleavage in both 4-RC_6_F_4_-Pd and Pd-Ar moieties. If the substituent Ar is the same for all *cis*-diarylpalladium, the rate of the intramolecular transformation from *cis*-(4-RC_6_F_4_)PdL_2_Ar to 4-RC_6_F_4_Ar should depend on the specific property of the 4-RC_6_F_4_ group. It may depend on the substituent electron parameters (SEP), σ_I_ and σ_R_°, of substituent R or 4-RC_6_F_4_ groups. We compared the relative rates of consumption C^rel^ (Table 3) of K[4-RC_6_F_4_BF_3_] with σ_I_ (R) and σ_R_° (R) (Table 5) and did not find any correlation between the obtained values.

**Table 5:** The substituent electron parameters of some R in RC_6_H_4_F (in CDCl_3_) [42].

R	H	Bu [43]	OCH_3_	Prz [44]	Pyr [44]	F	Im [44]

σ_I_	0	0.01	0.29	0.300	0.354	0.45	0.513
σ_R_°	0	−0.18	−0.56	−0.061	−0.210	−0.39	−0.155

SEP of the 4-RC_6_F_4_ groups are not reported as yet except inductive constants σ_I_ (C_6_F_5_) [43,45–46] and σ_I_ (2,3,5,6-C_6_F_4_H) [43], and resonance constants σ_R_° (C_6_F_5_) [45]. We bridge this gap using a series of biphenyls **4a–r** and **5a–r** and determine SEP of 4-RC_6_F_4_ groups using the Taft's method [42]. The ^19^F NMR spectra were measured in CHCl_3_ (non-polar weakly coordinating solvent) and in toluene (solvent for the present research). The calculated SEP values in both solvents are closely related to one another to indicate no specific intermolecular interaction biphenyl–solvent (Table 6) and obtained results are in agreement with the data by Sheppard for CCl_3_F or benzene [45]. When R = H, Bu, or alkoxy group, inductive constants σ_I_ (4-RC_6_F_4_) consist of 0.16–0.18. The 4-RC_6_F_4_ groups with R = F, pyrazol-1-yl, pyrrol-1-yl, indol-1-yl, imidazol-1-yl and benzimidazol-1-yl possess a higher electron-withdrawing effect (σ_I_ = 0.21 − 0.27) which achieves maximum for the 2,3,5,6-tetrafluoropyridyl group (σ_I_ = 0.35). Resonance constants σ_R_° of all tetrafluoroaryl groups are insignificant and reflect the substantial non-coplanarity of aryl moieties [45].

**Table 6:** The substituent electron parameters (SEP) of 4-RC_6_F_4_ groups.

R	σ_I_		σ_R_°	
	In toluene	In CHCl_3_	In toluene	In CHCl_3_

4-PhCH_2_O	0.160	0.172	0.010	0.014
4-iPrO	0.161		0.009	
4-PrO	0.163		0.008	
4-*t*-BuO	0.163	0.165	0.011	0.015
4-BuO	0.164	0.165	0.008	0.011
4-EtO	0.165	0.167	0.008	0.011
4-Bu	0.165	0.158	0.013	0.015
4-AllylO	0.166		0.010	
4-MeO	0.168		0.009	
4-PhCH_2_CH_2_O	0.170		0.008	
4-H^a^	0.179	0.193		0.029
4-PhO	0.201	0.211	0.021	0.023
4-Prz	0.214	0.253	0.033	0,037
4-Pyr	0.218	0.240	0.027	0.030
4-F^b^	0.220	0.235	0.022	0.025
4-Ind	0.235	0.250	0.032	0.034
4-Im	0.260		0.036	
4-Bim	0.270	0.293	0.040	0.044
4-CF_3_C_6_F_4_		0.300		0.056
4-C_5_NF_4_		0.346		0.064
2-C_5_NF_4_		0.248		0.070

^a^σ_I_ = 0.33 (water, 25 °C) [43]; ^b^σ_I_ = 0.31 (water, 25 °C) [43,46], 0.25 (CCl_3_F) [45]; σ_R_° = 0.02 (CCl_3_F) [45].

Unfortunately, the search for possible dependence between the relative rates of consumption of K[4-RC_6_F_4_BF_3_] and SEP of the respective 4-RC_6_F_4_ group does not show certain correlation.

## Conclusion

1. The relative reactivities of K[4-RC_6_F_4_BF_3_] in the Ag(I)-assisted Pd-catalyzed cross-coupling reactions with 3-IC_6_H_4_F, 4-IC_6_H_4_F, 4-IC_6_H_4_CH_3_ or 4-BrC_6_H_4_CH_3_ in the presence of P(*t*-Bu)_3_ or PPh_3_ (toluene, 100 °C, 8 h) differ negligibly from each other. The same results show the competitive cross-coupling of mixtures of K[4-RC_6_F_4_BF_3_] and K[C_6_F_5_BF_3_] (1:1, mol) with 4-BrC_6_H_4_CH_3_ at a low conversion of borates (<40%). K[2,3,5,6-C_5_NF_4_BF_3_] is extremely weakly nucleophilic and conversion in the cross-coupling product is no more than 5% in 8 h.

2. It is very likely that transmetallation of ArPdL*_n_*X with K[4-RC_6_F_4_BF_3_] proceeds on the surface of the silver(I) compounds (K[4-RC_6_F_4_BF_3_] and Ag*_m_*Y are insoluble in toluene). Neutral complex ArPdL*_n_*X is adsorbed on the acidic center (Ag^+^) of the solid surface to form highly reactive complex [ArPdL*_n_*···X···Ag*_m_*Y] which facilitates exchange of group BF_3_ in K[4-RC_6_F_4_BF_3_] by ArPdL*_n_* and thus accelerates the formation of (4-RC_6_F_4_)PdL*_n_*Ar.

3. Verification of the assumed correlation between reactivity (C^rel^) of K[4-RC_6_F_4_BF_3_] and the substituent electron parameters (SEP) (σ_I_ and σ_R_°) as the measure of the electron-withdrawing properties of 4-RC_6_F_4_ gives an unclear result. Intervals of change of both C^rel^ (0.6–1.0) and σ_I_ (0.16–0.27) are too narrow and small experimental errors of measurements may corrupt or mask electronic effect of 4-RC_6_F_4_ with respect to C_6_F_5_ group.

## Supporting Information

File 1 Full experimental details and compound characterization data.
