# Supplementary material for: Weakly nucleophilic potassium aryltrifluoroborates in palladium-catalyzed Suzuki–Miyaura reactions: relative reactivity of K[4-RC6F4BF3] and the role of silver-assistance in acceleration of transmetallation
Source: Beilstein J Org Chem. 2015 May 4;11:608–16. doi: 10.3762/bjoc.11.68 (PMC4464464; doi:10.3762/bjoc.11.68)
Supplement: File 1 — Full experimental details and compound characterization data. [file Beilstein_J_Org_Chem-11-608-s001.pdf]

Supporting Information

for

Weakly nucleophilic potassium aryltrifluoroborates in palladium-catalyzed Suzuki–Miyaura reactions: relative reactivity of $K[4\text{-}RC_6F_4BF_3]$ and the role of silver-assistance in acceleration of transmetallation

Vadim V. Bardin[1,2], Anton Yu. Shabalin[3] and Nicolay Yu. Adonin[2,3*]

Address: [1]N.N. Vorozhtsov Novosibirsk Institute of Organic Chemistry, SB RAS, Acad. Lavrentjev Ave. 9, Novosibirsk, 630090, Russian Federation, [2]Novosibirsk State University, Pirogova str., 2, Novosibirsk, 630090, Russian Federation and [3]G.K. Boreskov Institute of Catalysis, SB RAS, Acad. Lavrentjev Ave. 5, Novosibirsk, 630090, Russian Federation

Email: Nicolay Yu. Adonin* - adonin@catalysis.ru

*Corresponding author

Full experimental details and compound characterization data

Table of contents

1. General information. s2
2. Preparation of 2,3,5,6-$C_6F_4HOCH_2Ph$. s2
3. Preparation of 2,3,5,6-$C_6F_4HO(CH_2)_2Ph$. s2
4. Preparation of $K[4\text{-}Ph(CH_2)_2OC_6F_4BF_3]$ (1l). s3
5. Cross-coupling of $K[4\text{-}RC_6F_4BF_3]$ (1a-r) with 3-IC_6H_4F (2) or 3-IC_6H_4F (3). s3
6. Cross-coupling of $K[4\text{-}RC_6F_4BF_3]$ (1c-f,h-p) with 4-iodotoluene (9) or). 4-bromotoluene (11) s9
7. Cross-coupling of $K[4\text{-}RC_6F_4BF_3]$ (1b-p) and $K[C_6F_5BF_3]$(1a) (1:1) with 11. .... s12
8. Cross-coupling of 1a with 11 in the presence of Ag_nY. s13
9. Attempted reaction of 1a with K_2CO_3 and Ag_2O in toluene. s13
10. References. s13

1. General information.

The NMR spectra were acquired using a Bruker AVANCE 300 (^{1}H 300.13 MHz, ^{19}F 282.40 MHz) spectrometer. The chemical shifts are assigned to TMS (^{1}H), and CCl_3F (^{19}F, with C_6F_6 as secondary reference (-162.9 ppm)). High resolution mass spectra were acquired using a DFS spectrometer (EI mode, 70 eV). Palladium acetate (Fluka), silver oxide (Degussa), silver sulfate (Degussa), silver nitrate (Degussa), silver fluoride (Degussa), PPh_3 (Panreac), P(*t*-Bu)$_3$ (Aldrich), XPhos (Acros), and aryl halides (4-$BrC_6H_4CH_3$, 4-$IC_6H_4CH_3$, 3-IC_6H_4F, 4-IC_6H_4F) (Acros), NaH (60% dispersion in oil) (Sigma-Aldrich) were used as supplied. Silver tetrafluoroborate [1], borates K[4-$RC_6F_4BF_3$] (R = H [2], F [3], Bu, CH_2=$CHCH_2O$, EtO, MeO, *t*-BuO, BuO, PrO, iPrO, PhO [4], Pyr, Prz, Im, Bim, Ind [5]) and K[2,3,5,6-$C_5NF_4BF_3$] [6] were prepared as described elsewhere. Products 4'-$CH_3C_6H_4C_6F_5$ [7], 4'-$FC_6H_4C_6F_5$ [8], 3'-$FC_6H_4C_6F_5$ [9], 4'-$FC_6H_4C_6F_4H$ [10, 11], 1,2,4,5-tetrafluorobenzene [12], 2,3,5,6-C_6F_4HR (R = Bu [13], 4'-$CH_3C_6H_4$ [14], CH_2=$CHCH_2O$ [4], EtO [15], MeO [16], *t*-BuO [17], BuO [18], PrO [19], iPrO [20], PhO [21], Prz [22], Im [23, 24], Bim [5], Ind [25], Pyr [25]) were identified by ^{1}H and ^{19}F NMR spectra. Preparations of K[4-$PhCH_2CH_2OC_6F_4BF_3$], 2,3,5,6-$C_6F_4HOCH_2Ph$ and 2,3,5,6-$C_6F_4HOCH_2CH_2Ph$ are given below.

K_2CO_3 was calcined at 450 °C and stored into a glovebox before being used. Toluene was degassed three times by the freeze-pump-thaw method prior to use. Solids were weighed inside a glove box (less than 0.1 ppm O_2 and H_2O).

Abbreviations: pyrazol-1-yl (Prz), pyrrol-1-yl (Pyr), indol-1-yl (Ind), imidazol-1-yl (Im), benzimidazol-1-yl (Bim).

2. Preparation of 2,3,5,6-$C_6F_4HOCH_2Ph$.

K[4-$PhCH_2OC_6F_4BF_3$] (**1h**) (27 mg, 0.074 mmol) was stirred in MeOH (1 mL) into a sealed tube at 90 °C for 3 h. The ^{19}F NMR spectrum showed quantitative hydrodeboration of the substrate to 2,3,5,6-$C_6F_4HOCH_2Ph$ and signals of $[BF_4]^-$ and $[BF_3OMe]^-$. The solution was evaporated under reduced pressure at 40-50 °C (bath) and the residue was extracted with acetone. Solvent was removed on an evaporator to yield 2,3,5,6-$C_6F_4HOCH_2Ph$ (15 mg, 79%).

2,3,5,6-$C_6F_4HOCH_2Ph$: ^{1}H NMR (acetone-d$_6$): δ 7.5-7.4 (m, 5H, C_6H_5), 7.23 (m, 1H, H-4), 5.34 (s, 2H, CH_2O). ^{19}F NMR (acetone-d$_6$): δ –140.3 (ddd, 2F, $^3J_{FF}$ 21 Hz, $^5J_{FF}$ 9 Hz, $^3J_{FH}$ 10 Hz, F-3,5), –155.9 (ddd, 2F, $^3J_{FF}$ 21 Hz, $^5J_{FF}$ 9 Hz, $^4J_{FH}$ 7 Hz, F-2,6). HRMS (ESI) Calcd. for $C_{13}H_8F_4O$: 256.0511, found 256.0515.

3. Preparation of 2,3,5,6-$C_6F_4HO(CH_2)_2Ph$.

2,3,5,6-$C_6F_4HO(CH_2)_2Ph$ (13 mg, 79%) was prepared from K[4-$Ph(CH_2)_2OC_6F_4BF_3$] (**1l**, 23 mg, 0.061 mmol) in MeOH (1 mL) as 2,3,5,6-$C_6F_4HOCH_2Ph$.

2,3,5,6-$C_6F_4HO(CH_2)_2Ph$: ^{1}H NMR (acetone-d$_6$): δ 7.30 (m, 5H, C_6H_5), 7.20 (m, 1H, H-4), 4.50 (t, 2H, $^3J_{HH}$ 7 Hz, CH_2O), 3.11 (t, 2H, $^3J_{HH}$ 7 Hz, CH_2CH$_2$O). ^{19}F NMR (acetone-d$_6$): δ –140.3 (ddd, 2F, $^3J_{FF}$ 21 Hz, $^5J_{FF}$ 9 Hz, $^3J_{FH}$10 Hz, F-3,5), –156.7 (ddd, 2F, $^3J_{FF}$ 21 Hz, $^5J_{FF}$ 9 Hz, $^4J_{FH}$ 8 Hz, F-2,6). HRMS (ESI) Calcd. for $C_{14}H_{10}F_4O$: 270.0667, found 270.0670.

4. Preparation of K[4-$Ph(CH_2)_2OC_6F_4BF_3$] (1l).

A 50 mL flask was charged with NaH (596 mg, 14.9 mmol), DME (11 mL) and $PhCH_2CH_2OH$ (2.07 g, 17 mmol). The resulted suspension was stirred at 25 °C for 1 h under an atmosphere of argon, K[$C_6F_5BF_3$] (1.45 g, 5.3 mmol) was added in one portion and the reaction mixture was stirred for 4 h at 25 °C. Then K[HF_2] (8.31 g, 106 mmol), DME (11 mL) and MeCN (20 mL) were added, the suspension was stirred at 25 °C for 8 h, filtered through silica gel (40-60 μm) and the column was washed with MeCN (2x5 mL). The combined extract was evaporated to dryness to yield K[4-$Ph(CH_2)_2OC_6F_4BF_3$] (1.63 g, 82%).

K[4-$Ph(CH_2)_2OC_6F_4BF_3$] (1l): ^{1}H NMR (CD_3CN): δ 7.30 (m, 5H, C_6H_5), 4.34 (t, 2H, $^3J_{HH}$ 7 Hz, CH_2O), 3.03 (t, 2H, $^3J_{HH}$ 7 Hz, CH_2CH$_2$O). ^{19}F NMR (CD_3CN): δ –133.3 (q, 3F, $^1J_{BF}$ 40 Hz, BF_3), –136.4 (ddq, 2F, $^3J_{FF}$ 22 Hz, $^5J_{FF}$ 11 Hz, $^4J_{FF}$ 11 Hz, F-2,6), –159.2 (dd, 2F, $^3J_{FF}$ 24 Hz, $^5J_{FF}$ 11 Hz, F-3,5). Anal. calcd for $C_{14}H_9BF_7KO$ (376.12): C, 44.71; H, 2.41; F, 35.36; found: C, 44.1; H, 2.48; F, 37.6.

5. Cross-coupling of K[4-$RC_6F_4BF_3$] (1a-r) with 3-IC_6H_4F (2) or 3-IC_6H_4F (3).

A glass vessel equipped with a magnetic stir bar was flushed with dry argon, and charged with $Pd(OAc)_2$ (1.9 mg, 0.0084 mmol), PPh_3 (4.4 mg, 0.017 mmol), Ag_2O (23.2 mg, 0.100 mmol), K[4-$RC_6F_4BF_3$] (**1a-r**, 0.100 mmol), K_2CO_3 (23.1 mg, 0.167 mmol) and sealed with AluCap©. Toluene (1.0 mL) and fluoroiodobenzene (**2** or **3**, 18.5 mg, 0.084 mmol) were injected by syringe and the resulted suspension was stirred at 100 °C (bath) for 8 h. After cooling, $C_6H_5CF_3$ (10.0 μL, 0.082 mmol) (internal reference) was added, the suspension was filtered through silica gel (40-60 μm), the column was washed with toluene (2 mL) and volatiles were removed to dryness in an evaporator at 50-55 °C (bath) to give biphenyls **4a-r** or **5a-r** (viscous oil or white solid) (Table 1). When R = Im or Bim, phosphine XPhos (0.017 mmol) was used instead of PPh_3.

Reaction of K[2,3,5,6-$C_6HF_4BF_3$] (**1b**) and **2** gave 2,3,3',5,6-pentafluorobiphenyl (**4b**) (0.026 mmol) and hexafluoro-*para*-terphenyl 1,4-(3'-$FC_6H_4)_2C_6F_4$ (0.011 mmol). Similarly, 2,3,4',5,6-

pentafluorobiphenyl **5b** (0.024 mmol) and hexafluoro-*para*-terphenyl 1,4-(4'-$FC_6H_4)_2C_6F_4$ (0.012 mmol) were obtained from **1b** and **3**. A mixture of these biphenyls with *para*-terphenyls was not separated and constitution of products was proved by ^{19}F NMR and HRMS.

A suspension derived from K[4-Bim$C_6F_4BF_3$] (**1r**) was filtered through silica gel (40-60 µm), the column was washed with toluene (2 mL) and with $CHCl_3$. The extract in $CHCl_3$ was evaporated under reduced pressure at 50-55 °C (bath) to give **4r** (white solid, 0.020 mmol) or **5r** (white solid, 0.020 mmol) (both were contaminated with 2,3,5,6-C_6F_4HBim). Biphenyls **4q** (0.010 mmol) and **5q** (0.012 mmol) were obtained in a mixture with known 2,3,5,6-C_6F_4HIm.

Attempts to prepare phenylpyridines 4-(3'-FC_6H_4)C_5NF_4 and 4-(4'-FC_6H_4)C_5NF_4 similar way led to very low yield of products (2 and 5%, respectively).

1-Methoxy-2,3,3',5,6-pentafluorobiphenyl (4d): ^{19}F NMR (toluene): δ –112.1 (m, 1F, F-3'), –145.2 (dd, 2F, $^3J_{FF}$ 22.2 Hz, $^5J_{FF}$ 8.4 Hz, F-3,5), –158.2 (dd, 2F, $^3J_{FF}$ 22.4 Hz, $^5J_{FF}$ 8.2 Hz, F-2,6). HRMS calcd for $C_{13}H_7F_5O$: 274.0417; found: 274.0417.

1-Ethoxy-2,3,3',5,6-pentafluorobiphenyl (4e): ^{1}H NMR ($CDCl_3$): δ 7.4-7.1 (m, 4H), 4.34 (q, 2H, $^3J_{HH}$ 7 Hz, OCH_2), 1.44 (t, 3H, $^3J_{HH}$ 7 Hz, CH_3). ^{19}F NMR ($CDCl_3$): δ –113.31 (td, 1F, $^3J_{FH}$ 8.8 Hz, $^4J_{FH}$ 6.2 Hz, F-3), –145.9 (dd, 2F, $^3J_{FF}$ 22.0 Hz, $^5J_{FF}$ 8.6 Hz, F-3,5), –158.0 (dd, 2F, $^3J_{FF}$ 22 Hz, $^5J_{FF}$ 9 Hz, F-2,6). ^{19}F NMR (toluene): δ –112.1 (m, 1F, F-3'), –145.2 (dd, 2F, $^3J_{FF}$ 22.0 Hz, $^5J_{FF}$ 8.6 Hz, F-3,5), –157.5 (dd, 2F, $^3J_{FF}$ 21.8 Hz, $^5J_{FF}$ 8.4 Hz, F-2,6). HRMS calcd for $C_{14}H_9F_5O$: 288.0574; found: 288.0574.

1-Propoxy-2,3,3',5,6-pentafluorobiphenyl (4f): ^{19}F NMR (toluene): δ –112.1 (m, 1F, F-3'), –145.3 (dd, 2F, $^3J_{FF}$ 22.2 Hz, $^5J_{FF}$ 8.4 Hz, F-3,5), –157.5 (dd, 2F, $^3J_{FF}$ 22.2 Hz, $^5J_{FF}$ 8.4 Hz, F-2,6). HRMS calcd for $C_{15}H_{11}F_5O$: 302.0730; found: 302.0730.

1-Isoropoxy-2,3,3',5,6-pentafluorobiphenyl (4j): ^{19}F NMR (toluene): δ –112.1 (m, 1F, F-3'), –145.9 (dd, 2F, $^3J_{FF}$ 22.4 Hz, $^5J_{FF}$ 8.6 Hz, F-3,5), –156.4 (dd, 2F, $^3J_{FF}$ 22.2 Hz, $^5J_{FF}$ 8.8 Hz, F-2,6). HRMS calcd for $C_{15}H_{11}F_5O$: 302.0730; found: 302.0730.

1-Butoxy-2,3,3',5,6-pentafluorobiphenyl (4i): ^{1}H NMR ($CDCl_3$): δ 7.4-7.0 (m, 4H), 4.27 (t, 2H, $^3J_{HH}$ 6 Hz, OCH_2), 1.78, (m, 2H), 1.52 (m, 2H), 0.98 (t, 3H, $^3J_{HH}$ 7 Hz, CH_3). ^{19}F NMR ($CDCl_3$): δ –113.33 (m, 1F, F-3'), –146.0 (dd, 2F, $^3J_{FF}$ 22 Hz, $^5J_{FF}$ 8 Hz, F-3,5), –158.0 (dd, 2F, $^3J_{FF}$ 22 Hz, $^5J_{FF}$ 8 Hz, F-2,6). ^{19}F NMR (toluene): δ –112.1 (m, 1F, F-3'), –145.2 (dd, 2F, $^3J_{FF}$ 22.2 Hz, $^5J_{FF}$ 8.4 Hz, F-3,5), –157.5 (dd, 2F, $^3J_{FF}$ 22.2 Hz, $^5J_{FF}$ 8.4 Hz, F-2,6). HRMS calcd for $C_{16}H_{13}F_5O$: 316.0887; found: 316.0888.

1-*tert*-Butoxy-2,3,3',5,6-pentafluorobiphenyl (4k): ^{1}H NMR ($CDCl_3$): δ 7.6-7.1 (m, 4H), 1.43 (s, 9H, $OCMe_3$). ^{19}F NMR ($CDCl_3$): δ –113.33 (td, 1F, $^3J_{FH}$ 9 Hz, $^4J_{FH}$ 6.1 Hz, F-3'), –146.2 (dd, 2F, $^3J_{FF}$ 23 Hz, $^5J_{FF}$ 9.1 Hz, F-3,5), –152.3 (dd, 2F, $^3J_{FF}$ 23 Hz, $^5J_{FF}$ 9.1 Hz, F-2,6). ^{19}F NMR (toluene): δ –112.1 (m, 1F, F-3'), –145.3 (dd, 2F, $^3J_{FF}$ 22.9 Hz, $^5J_{FF}$ 9.1 Hz, F-3,5), –151.7 (dd, 2F, $^3J_{FF}$ 22.8 Hz, $^5J_{FF}$ 9.1 Hz, F-2,6). HRMS calcd for $C_{15}H_{10}F_5O$: 301.0646 (m/z –CH_3); found: 301.0649.

1-Benzyloxy-2,3,3',5,6-pentafluorobiphenyl (4h): ^{1}H NMR ($CDCl_3$): δ 7.7-6.9 (m, 9H), 5.14 (s, 2H, CH_2). ^{19}F NMR ($CDCl_3$): δ –113.28 (td, 1F, $^3J_{FH}$ 9 Hz, $^4J_{FH}$ 6.2 Hz, F-3'), –145.7 (dd, 2F, $^3J_{FF}$ 22.4 Hz, $^5J_{FF}$ 8.6 Hz, F-3,5), –156.9 (dd, 2F, $^3J_{FF}$ 22.2 Hz, $^5J_{FF}$ 8.6 Hz, F-2,6). ^{19}F NMR (toluene): δ –112.1 (m, 1F, F-3'), –145.1 (dd, 2F, $^3J_{FF}$ 22.4 Hz, $^5J_{FF}$ 8.6 Hz, F-3,5), –156.4 (dd, 2F, $^3J_{FF}$ 22.2 Hz, $^5J_{FF}$ 8.4 Hz, F-2,6). HRMS calcd for $C_{19}H_{11}F_5O$: 350.0725; found: 350.0722.

1-(2-Phenylethoxy)-2,3,3',5,6-pentafluorobiphenyl (4l): ^{19}F NMR (toluene): δ –112.1 (m, 1F, F-3'), –145.2 (dd, 2F, $^3J_{FF}$ 22.0 Hz, $^5J_{FF}$ 8.6 Hz, F-3,5), –157.2 (dd, 2F, $^3J_{FF}$ 22.0 Hz, $^5J_{FF}$ 8.6 Hz, F-2,6). HRMS calcd for $C_{20}H_{13}F_5O$: 364.0887; found: 364.0887.

1-Allyloxy-2,3,3',5,6-pentafluorobiphenyl (4c): ^{19}F NMR (toluene): δ –112.1 (m, 1F, F-3'), –145.2 (dd, 2F, $^3J_{FF}$ 22.2 Hz, $^5J_{FF}$ 8.8 Hz, F-3,5), –156.7 (dd, 2F, $^3J_{FF}$ 22.0 Hz, $^5J_{FF}$ 8.6 Hz, F-2,6). HRMS calcd for $C_{15}H_9F_5O$: 300.0574; found: 300.0574.

1-Phenoxy-2,3,3',5,6-pentafluorobiphenyl (4m): ^{19}F NMR ($CDCl_3$): δ –113.0 (td, 1F, $^3J_{FH}$ 8.9 Hz, $^4J_{FH}$ 6.1 Hz, F-3'), –144.4 (dd, 2F, $^3J_{FF}$ 22.4 Hz, $^5J_{FF}$ 9.4 Hz, F-3,5), –154.9 (dd, 2F, $^3J_{FF}$ 22.4 Hz, $^5J_{FF}$ 9.4 Hz, F-2,6). ^{19}F NMR (toluene): δ –111.8 (m, 1F, F-3'), –143.8 (dd, 2F, $^3J_{FF}$ 22.4 Hz, $^5J_{FF}$ 9.5 Hz, F-3,5), –154.4 (dd, 2F, $^3J_{FF}$ 22.4 Hz, $^5J_{FF}$ 9.1 Hz, F-2,6). HRMS calcd for $C_{18}H_9F_5O$: 336.0573; found: 336.0572.

1-Pyrrol-1-yl-2,3,3',5,6-pentafluorobiphenyl (4o): ^{19}F NMR ($CDCl_3$): δ –112.8 (tt, 1F, $^3J_{FH}$ 9 Hz, $^4J_{FH}$ 6 Hz, F-3'), –143.7 (dd, 2F, $^3J_{FF}$ 22 Hz, $^5J_{FF}$ 10 Hz, F-3,5), –150.6 (dd, 2F, $^3J_{FF}$ 22 Hz, $^5J_{FF}$ 10 Hz, F-2,6). ^{19}F NMR (toluene): δ –111.7 (m, 1F, F-3'), –143.5 (dd, 2F, $^3J_{FF}$ 22.4 Hz, $^5J_{FF}$ 9.9 Hz, F-3,5), –150.3 (dd, 2F, $^3J_{FF}$ 22.4 Hz, $^5J_{FF}$ 9.9 Hz, F-2,6). HRMS calcd for $C_{16}H_8F_5N$: 309.0577; found: 309.0574.

1-Pyrazol-1-yl-2,3,3',5,6-pentafluorobiphenyl (4p): ^{19}F NMR ($CDCl_3$): δ –112.70 (td, 1F, $^3J_{FH}$ 9 Hz, $^4J_{FH}$ 6 Hz, F-3'), –143.3 (dd, 2F, $^3J_{FF}$ 21.0 Hz, $^5J_{FF}$ 10 Hz, F-3,5), –149.1 (dd, 2F, $^3J_{FF}$ 21

Hz, $^5J_{FF}$ 10 Hz, F-2,6). ^{19}F NMR (toluene): δ –111.8 (m, 1F, F-3'), –143.4 (dd, 2F, $^3J_{FF}$ 22.0 Hz, $^5J_{FF}$ 9.9 Hz, F-3,5), –148.7 (dd, 2F, $^3J_{FF}$ 21.8 Hz, $^5J_{FF}$ 9.7 Hz, F-2,6). HRMS calcd for $C_{15}H_7F_5N_2$: 310.0529; found: 310.0526.

1-Indol-1-yl-2,3,3',5,6-pentafluorobiphenyl (4n): ^{19}F NMR ($CDCl_3$): δ –112.73 (td, 1F, $^3J_{FH}$ 8.5 Hz, $^4J_{FH}$ 6.4 Hz, F-3'), –143.3 (dd, 2F, $^3J_{FF}$ 22.0 Hz, $^5J_{FF}$ 9.0 Hz, F-3,5), –147.1 (dd, 2F, $^3J_{FF}$ 21 Hz, $^5J_{FF}$ 9 Hz, F-2,6).^{19}F NMR (toluene): δ –111.6 (m, 1F, F-3'), –142.9 (dd, 2F, $^3J_{FF}$ 22.2 Hz, $^5J_{FF}$ 10.1 Hz, F-3,5), –146.3 (dd, 2F, $^3J_{FF}$ 22.6 Hz, $^5J_{FF}$ 10.1 Hz, F-2,6). HRMS calcd for $C_{20}H_{10}F_5N$: 359.0728; found: 359.0733.

1-Benzimidazolyl-2,3,3',5,6-pentafluorobiphenyl (4r): ^{19}F NMR ($CDCl_3$): δ –112.73 (td, 1F, $^3J_{FH}$ 8.8 Hz, $^4J_{FH}$ 6.1 Hz, F-3'), –142.0 (m, 2F, F-3,5), –146.5 (m, 2F, F-2,6). ^{19}F NMR (toluene): δ –111.3 (m, 1F, F-3'), –142.1 (dd, 2F, $^3J_{FF}$ 21.3 Hz, $^5J_{FF}$ 9.3 Hz, F-3,5), –146.2 (dd, 2F, $^3J_{FF}$ 21.3 Hz, $^5J_{FF}$ 9.3 Hz, F-2,6). HRMS calcd for $C_{19}H_9F_5N_2$: 360.0685; found: 360.0689.

1-Butyl-2,3,3',5,6-pentafluorobiphenyl (4g): ^{1}H NMR ($CDCl_3$): δ 7.3-7.0 (m, 4H), 2.64 (t, 2H, $^3J_{HH}$ 8 Hz, CH_2), 1.42-1.30 (m, 4H, 2CH_2), 0.94 (t, 3H, $^3J_{HH}$ 7 Hz, CH_3). ^{19}F NMR ($CDCl_3$): δ –113.38 (td, 1F, $^3J_{FH}$ 9 Hz, $^4J_{FH}$ 6 Hz, F-3'), –145.7 and –146.0 (m, 4F, F-2,3,5,6). ^{19}F NMR (toluene): δ –112.1 (m, 1F, F-3'), –144.9 (m, 2F, F-3,5), –145.0 (m, 2F, F-2,6). HRMS calcd for $C_{16}H_{13}F_5$: 300.0932; found: 300.0923.

2,3,3',5,6-Pentafluorobiphenyl (4b): ^{19}F NMR ($CDCl_3$): δ –113.13 (td, 1F, $^3J_{FH}$ 8.8 Hz, $^4J_{FH}$ 6 Hz, F-3'), –139.5 (ddd, 2F, $^3J_{FF}$ 22 Hz, $^5J_{FF}$ 9 Hz, $^3J_{FH}$ 13 Hz, F-2,6), –144.4 (ddd, 2F, $^3J_{FF}$ 22 Hz, $^5J_{FF}$ 9 Hz, $^3J_{FH}$ 13 Hz, F-3,5). ^{19}F NMR (toluene): δ –112.0 (m, 1F, F-3'), –138.9 (m, 4F, F-2,6), –143.9 (m; 2F; F-3,5). HRMS calcd for $C_{12}H_5F_5$: 244.0311; found: 244.0312.

2,3,3',4,5,6-Hexafluorobiphenyl (4a): ^{19}F NMR (toluene): δ –111.7 (td, 1F, $^3J_{FH}$ 8.9 Hz, $^4J_{FH}$ 5.9 Hz, F-3'), –143.7 (dd, 2F, $^3J_{FF}$ 23.1 Hz, $^5J_{FF}$ 8.0 Hz, F-3,5), –155.2 (t, 1F, $^3J_{FF}$ 21.6 Hz, F-4), –162.3 (td, 2F, $^3J_{FF}$ 22.2 Hz, $^5J_{FF}$ 7.1 Hz, F-2,6).

1-Methoxy-2,3,4',5,6-pentafluorobiphenyl (5d): ^{19}F NMR (Toluene): δ –111.8 (tt, 1F, $^3J_{FH}$ 8.7 Hz, $^4J_{FH}$ 5.0 Hz, F-4'), –145.6 (dd, 2F, $^3J_{FF}$ 22.0 Hz, $^5J_{FF}$ 8.6 Hz, F-3,5), –158.3 (dd, 2F, $^3J_{FF}$ 22.0 Hz, $^5J_{FF}$ 8.6 Hz, F-2,6). HRMS calcd for $C_{13}H_7F_5O$: 274.0417; found: 274.0417.

1-Ethoxy-2,3,4',5,6-pentafluorobiphenyl (5e): ^{1}H NMR ($CDCl_3$): δ 7.4-7.0 (m, 4H), 4.34 (q, 2H, $^3J_{HH}$ 7 Hz, OCH_2), 1.45 (t, 3H, $^3J_{HH}$ 7 Hz, CH_3). ^{19}F NMR ($CDCl_3$): δ –112.98 (tt, 1F, $^3J_{FH}$

8.7 Hz, $^4J_{FH}$ 5.0 Hz, F-4'), –146.3 (dd, 2F, $^3J_{FF}$ 22 Hz, $^5J_{FF}$ 8.6 Hz, F-3,5), –158.2 (dd, 2F, $^3J_{FF}$ 22 Hz, $^5J_{FF}$ 8.6 Hz, F-2,6). ^{19}F NMR (toluene): δ –111.9 (tt, 1F, $^3J_{FH}$ 8.5 Hz, $^4J_{FH}$ 5.2 Hz, F-4'), –145.6 (dd, 2F, $^3J_{FF}$ 22.2 Hz, $^5J_{FF}$ 8.8 Hz, F-3,5), –157.6 (dd, 2F, $^3J_{FF}$ 22.4 Hz, $^5J_{FF}$ 8.6 Hz, F-2,6). HRMS calcd for $C_{14}H_9F_5O$: 288.0574; found: 288.0574.

1-Propoxy-2,3,4',5,6-pentafluorobiphenyl (5f): ^{19}F NMR (toluene): δ –111.9 (tt, 1F, $^3J_{FH}$ 8.6 Hz, $^4J_{FH}$ 5.2 Hz, F-4'), –145.6 (dd, 2F, $^3J_{FF}$ 22.6 Hz, $^5J_{FF}$ 8.8 Hz, F-3,5), –157.6 (dd, 2F, $^3J_{FF}$ 22.4 Hz, $^5J_{FF}$ 8.6 Hz, F-2,6). HRMS calcd for $C_{15}H_{11}F_5O$: 302.0730; found: 302.0731.

1-Isoropoxy-2,3,4',5,6-pentafluorobiphenyl (5j): ^{19}F NMR (toluene): δ –111.9 (tt, 1F, $^3J_{FH}$ 8.9 Hz, $^4J_{FH}$ 4.9 Hz, F-4'), –145.6 (dd, 2F, $^3J_{FF}$ 22.6 Hz, $^5J_{FF}$ 8.8 Hz, F-3,5), –156.5 (dd, 2F, $^3J_{FF}$ 22.4 Hz, $^5J_{FF}$ 9.1 Hz, F-2,6). HRMS calcd for $C_{15}H_{11}F_5O$: 302.0730; found: 302.0730.

1-Butoxy-2,3,4',5,6-pentafluorobiphenyl (5i): ^{1}H NMR ($CDCl_3$): δ 7.4-7.0 (m, 4H), 4.25 (t, 2H, $^3J_{HH}$ 6 Hz, OCH_2), 1.77, (m, 2H), 1.52 (m, 2H), 0.98 (t, 3H, $^3J_{HH}$ 7 Hz, CH_3). ^{19}F NMR ($CDCl_3$) –113.01 (tt, 1F, $^3J_{FH}$ 9 Hz, $^4J_{FH}$ 5 Hz, F-4'), –146.3 (dd, 2F, $^3J_{FF}$ 22 Hz, $^5J_{FF}$ 9 Hz, F-3,5), –158.2 (dd, 2F, $^3J_{FF}$ 22 Hz, $^5J_{FF}$ 9 Hz, F-2,6). ^{19}F NMR (toluene): δ –111.9 (tt, 1F, $^3J_{FH}$ 8.9 Hz, $^4J_{FH}$ 4.9 Hz, F-4'), –145.6 (dd, 2F, $^3J_{FF}$ 22.2 Hz, $^5J_{FF}$ 8.8 Hz, F-3,5), –157.6 (dd, 2F, $^3J_{FF}$ 22.4 Hz, $^5J_{FF}$ 8.6 Hz, F-2,6). HRMS calcd for $C_{16}H_{13}F_5O$: 316.0887; found: 316.0888.

1-*tert*-Butoxy-2,3,4',5,6-pentafluorobiphenyl (5k): ^{1}H NMR ($CDCl_3$): δ 7.6-7.1 (m, 4H), 1.43 (s, 9H, $OCMe_3$). ^{19}F NMR ($CDCl_3$): δ –112.9 (tt, 1F, $^3J_{FH}$ 8.5 Hz, $^4J_{FH}$ 5.2 Hz, F-4'), –146.6 (dd, 2F, $^3J_{FF}$ 22.9 Hz, $^5J_{FF}$ 8.6 Hz, F-3,5), –152.5 (dd, 2F, $^3J_{FF}$ 23.3 Hz, $^5J_{FF}$ 9.1 Hz, F-2,6). ^{19}F NMR (toluene): δ –111.8 (tt, 1F, $^3J_{FH}$ 8.5 Hz, $^4J_{FH}$ 5.3 Hz, F-4'), –145.7 (dd, 2F, $^3J_{FF}$ 22.9 Hz, $^5J_{FF}$ 8.6 Hz, F-3,5), –151.9 (dd, 2F, $^3J_{FF}$ 23.3 Hz, $^5J_{FF}$ 9.1 Hz, F-2,6). HRMS calcd for $C_{15}H_{10}F_5O$: 301.0646 (m/z –CH_3); found: 301.0641.

1-(2-Phenylethoxy)-2,3,4',5,6-pentafluorobiphenyl (5l): ^{19}F NMR (toluene): δ –111.8 (tt, 1F, $^3J_{FH}$ 8.5 Hz, $^4J_{FH}$ 5.2 Hz, F-4'), –145.6 (dd, 2F, $^3J_{FF}$ 22.4 Hz, $^5J_{FF}$ 8.6 Hz, F-3,5), –157.3 (dd, 2F, $^3J_{FF}$ 22.0 Hz, $^5J_{FF}$ 8.6 Hz, F-2,6). HRMS calcd for $C_{20}H_{13}F_5O$: 364.0887; found: 364.0887.

1-Benzyloxy-2,3,4',5,6-pentafluorobiphenyl (5h): ^{1}H NMR ($CDCl_3$): δ 7.7-6.9 (m, 9H), 5.14 (s, 2H, CH_2). ^{19}F NMR ($CDCl_3$): δ –112.88 (tt, 1F, $^3J_{FH}$ 8.6 Hz, $^4J_{FH}$ 5.2 Hz, F-4'), –146.1 (dd, 2F, $^3J_{FF}$ 22.6 Hz, $^5J_{FF}$ 8.6 Hz, F-3,5), –157.1 (dd, 2F, $^3J_{FF}$ 22.0 Hz, $^5J_{FF}$ 8.6 Hz, F-2,6). ^{19}F NMR (toluene): δ –111.9 (tt, 1F, $^3J_{FH}$ 8.8 Hz, $^4J_{FH}$ 4.9 Hz, F-4'), –145.5 (dd, 2F, $^3J_{FF}$ 22.6 Hz, $^5J_{FF}$ 8.8 Hz, F-3,5), –156.5 (dd, 2F, $^3J_{FF}$ 22.6 Hz, $^5J_{FF}$ 8.8 Hz, F-2,6). HRMS calcd for $C_{19}H_{11}F_5O$:

350.0725; found: 350.0723.

1-Allyloxy-2,3,4',5,6-pentafluorobiphenyl (5c): ^{19}F NMR (toluene): δ –111.8 (m, 1F, F-4'), –145.5 (dd, 2F, $^3J_{FF}$ 22.6 Hz, $^5J_{FF}$ 8.8 Hz, F-3,5), –156.9 (dd, 2F, $^3J_{FF}$ 22.4 Hz, $^5J_{FF}$ 8.6 Hz, F-2,6). HRMS calcd for $C_{15}H_9F_5O$: 300.0574; found: 300.0574.

1-Phenoxy-2,3,4',5,6-pentafluorobiphenyl (5m): ^{1}H NMR ($CDCl_3$): δ 7.8-6.9 (m, 9H). ^{19}F NMR ($CDCl_3$): δ –112.33 (tt, 1F, $^3J_{FH}$ 8.7 Hz, $^4J_{FH}$ 5 Hz, F-4'), –144.8 (dd, 2F, $^3J_{FF}$ 22 Hz, $^5J_{FF}$ 9 Hz, F-3,5), –155.2 (dd, 2F, $^3J_{FF}$ 22 Hz, $^5J_{FF}$ 9 Hz, F-2,6). ^{19}F NMR (toluene): δ –111.2 (tt, 1F, $^3J_{FH}$ 8.5 Hz, $^4J_{FH}$ 5.5 Hz, F-4'), –144.2 (dd, 2F, $^3J_{FF}$ 22.9 Hz, $^5J_{FF}$ 9.5 Hz, F-3,5), –154.6 (dd, 2F, $^3J_{FF}$ 22.9 Hz, $^5J_{FF}$ 9.5 Hz, F-2,6). HRMS calcd for $C_{18}H_9F_5O$: 336.0573; found: 336.0574.

1-Pyrrol-1-yl-2,3,4',5,6-pentafluorobiphenyl (5o): ^{19}F NMR ($CDCl_3$): δ –111.9 (tt, 1F, $^3J_{FH}$ 8.5 Hz, $^4J_{FH}$ 5.1 Hz, F-4'), –144.1 (dd, 2F, $^3J_{FF}$ 22 Hz, $^5J_{FF}$ 10 Hz, F-3,5), –150.8 (dd, 2F, $^3J_{FF}$ 22 Hz, $^5J_{FF}$ 10 Hz, F-2,6). ^{19}F NMR (toluene): δ –111.9 (tt, 1F, $^3J_{FH}$ 8.2 Hz, $^4J_{FH}$ 5.0 Hz, F-4'), –143.9 (dd, 2F, $^3J_{FF}$ 22.6 Hz, $^5J_{FF}$ 9.7 Hz, F-3,5), –150.5 (dd, 2F, $^3J_{FF}$ 22.6 Hz, $^5J_{FF}$ 10.1 Hz, F-2,6). HRMS calcd for $C_{16}H_8F_5N$: 309.0577; found: 309.0574.

1-Pyrazol-1-yl-2,3,4',5,6-pentafluorobiphenyl (5p): ^{19}F NMR ($CDCl_3$): δ –111.60 (tt, 1F, $^3J_{FH}$ 8.5 Hz, $^4J_{FH}$ 5 Hz, F-4'), –143.7 (dd, 2F, $^3J_{FF}$ 21.5 Hz, $^5J_{FF}$ 10 Hz, F-3,5), –149.4 (dd, 2F, $^3J_{FF}$ 22 Hz, $^5J_{FF}$ 10 Hz, F-2,6). ^{19}F NMR (toluene): δ –110.8 (tt, 1F, $^3J_{FH}$ 8.5 Hz, $^4J_{FH}$ 4.7 Hz, F-4'), –143.8 (dd, 2F, $^3J_{FF}$ 21.8 Hz, $^5J_{FF}$ 9.3 Hz, F-3,5), –148.9 (dd, 2F, $^3J_{FF}$ 21.6 Hz, $^5J_{FF}$ 9.5 Hz, F-2,6). HRMS calcd for $C_{15}H_7F_5N_2$: 310.0529; found: 310.0530.

1-Indol-1-yl-2,3,4',5,6-pentafluorobiphenyl (5n): ^{19}F NMR ($CDCl_3$): δ –111.74 (tt, 1F, $^3J_{FH}$ 8.5 Hz, $^4J_{FH}$ 4.8 Hz, F-4'), –143.8 (dd, 2F, $^3J_{FF}$ 21.0 Hz, $^5J_{FF}$ 9.0 Hz, F-3,5), –147.1 (dd, 2F, $^3J_{FF}$ 21 Hz, $^5J_{FF}$ 9 Hz, F-2,6). ^{19}F NMR (toluene): δ –112.7 (tt, 1F, $^3J_{FH}$ 9.5 Hz, $^4J_{FH}$ 5.3 Hz, F-4'), –143.4 (dd, 2F, $^3J_{FF}$ 22.0 Hz, $^5J_{FF}$ 9.5 Hz, F-3,5), –146.5 (dd, 2F, $^3J_{FF}$ 22.6 Hz, $^5J_{FF}$ 10.6 Hz, F-2,6). HRMS calcd for $C_{20}H_{10}F_5N$: 359.0728; found: 359.0723.

1-Benzimidazolyl-2,3,4',5,6-pentafluorobiphenyl (5r): ^{19}F NMR ($CDCl_3$): δ –112.73 (td, 1F, $^3J_{FH}$ 8.8 Hz, $^4J_{FH}$ 6.1 Hz, F-3'), –142.0 (m, 2F, F-3,5), –146.5 (m, 2F, F-2,6). ^{19}F NMR (toluene): δ –110.2 (m, 1F, F-4'), –142.6 (dd, 2F, $^3J_{FF}$ 21.3 Hz, $^5J_{FF}$ 9.3 Hz, F-3,5), –146.4 (dd, 2F, $^3J_{FF}$ 21.3 Hz, $^5J_{FF}$ 9.3 Hz, F-2,6). HRMS calcd for $C_{19}H_9F_5N_2$: 360.0685; found: 360.0687.

1-Butyl-2,3,4',5,6-pentafluorobiphenyl (5g): ^{19}F NMR ($CDCl_3$): δ –112.94 (tt, 1F, $^3J_{FH}$ 9 Hz,

$^4J_{FH}$ 5 Hz, F-4'), –146.0 and –146.4 (m, 4F, F-2,3,5,6). ^{19}F NMR (toluene): δ –111.7 (m, 1F, F-4'), –145.3 (m, 4F, F-2,3,5,6). HRMS calcd for $C_{16}H_{13}F_5$: 300.0932; found: 300.0938.

2,3,4',5,6-Pentafluorobiphenyl (5b): ^{19}F NMR ($CDCl_3$): δ –112.27 (td, 1F, $^3J_{FH}$ 8.6 Hz, $^4J_{FH}$ 5.2 Hz, F-4'), –139.8 (ddd, 2F, $^3J_{FF}$ 22 Hz, $^5J_{FF}$ 9 Hz, $^3J_{FH}$ 13 Hz, F-2,6), –144.8 (ddd, 2F, $^3J_{FF}$ 22 Hz, $^5J_{FF}$ 9 Hz, $^3J_{FH}$ 13 Hz, F-3,5).^{19}F NMR (toluene): δ –111.3 (m, 1F, F-4'), –139.1 (m, 4F, F-2,6), –144.2 (m; 2F; F-3,5).

2,3,4,4',5,6-Hexafluorobiphenyl (5a): ^{19}F NMR (toluene): δ –111.1 (tt, 1F, $^3J_{FH}$ 8.4 Hz, $^4J_{FH}$ 5.2 Hz, F-4'), –144.1 (dd, 2F, $^3J_{FF}$ 23.3 Hz, $^5J_{FF}$ 7.8 Hz, F-3,5), –155.9 (t, 1F, $^3J_{FF}$ 21.6 Hz, F-4), –162.5 (td, 2F, $^3J_{FF}$ 22.4 Hz, $^5J_{FF}$ 7.3 Hz, F-2,6).

Hexafluoro-*para*-terphenyl 1,4-(3'-$FC_6H_4)_2C_6F_4$ (in mixture with 2,3,3',5,6-pentafluorobiphenyl**)**. ^{19}F NMR ($CDCl_3$): δ –113.0 (td, 2F, $^3J_{FH}$ 8.8 Hz, $^4J_{FH}$ 6.2 Hz, F-3'), –144.4 (s, 4F, F-2,3,5,6). HRMS calcd for $C_{18}H_8F_6$: 338.05304; found: 338.0533.

Hexafluoro-*para*-terphenyl 1,4-(4'-$FC_6H_4)_2C_6F_4$ (in mixture with 2,3,4',5,6-pentafluorobiphenyl**)**. ^{19}F NMR ($CDCl_3$): δ –112.35 (tt, 2F, $^3J_{FH}$ 8.5 Hz, $^4J_{FH}$ 5.2 Hz, F-4'), –145.1 (s, 4F, F-2,3,5,6). HRMS calcd for $C_{18}H_8F_6$: 338.05304; found: 338.0533.

6. Cross-coupling of K[4-$RC_6F_4BF_3$] (1c-f,h-p) with 4-iodotoluene (9) or 4-bromotoluene (11).

A glass vessel equipped with a magnetic stir bar was flushed with dry argon, and charged with $Pd(OAc)_2$ (1.1 mg, 0.005 mmol), PPh_3 (2.6 mg, 0.010 mmol), 4-iodotoluene (**9**, 21.8 mg, 0.10 mmol), Ag_2O (27.8 mg, 0.12 mmol), K[4-$RC_6F_4BF_3$] (0.12 mmol) (R = MeO (**1d**), EtO (**1e**), PrO (**1f**), *i*-PrO (**1j**), *t*-BuO (**1k**), $PhCH_2O$ (**1h**), PhO (**1m**), Pyr (**1o**)), K_2CO_3 (27 mg, 0.20 mmol) and toluene (1 mL). The vessel was sealed with AluCap©, the resulted suspension was stirred for 8 h at 100 °C (bath), and cooled to 25 °C. The black suspension was diluted with toluene (2 mL), filtered through silica gel (40-60 μm), the column was washed with toluene (1 mL) and the combined solution was evaporated under reduced pressure to form biphenyls **10d-f,h,j,k,m,o**. In the case of **1h** benzyl 2,3,5,6-tetrafluorophenyl ether (32%) was detected too (Table 2).
Cross-coupling of K[4-$RC_6F_4BF_3$] (0.200 mmol) (R = BuO (**1i**), $PhCH_2CH_2O$ (**1l**), Prz (**1p**), Ind (**1n**), CH_2=$CHCH_2O$ (**1c**)) with 4-bromotoluene (**11**, 28.6 mg, 0.167 mmol) was performed in similar manner using $Pd(OAc)_2$ (1.9 mg, 0.008 mmol), P(*t*-Bu)$_3$ (3.4 mg, 0.017 mmol), Ag_2O (46.4 mg, 0.200 mmol), K_2CO_3 (46 mg, 0.334 mmol) and toluene (1 mL) to give 4-(4'-

$CH_3C_6H_4)C_6F_4R$. In the case of **1c** allyl 2,3,5,6-tetrafluorophenyl ether (28% yield) was detected too (Table 2).

1-Methoxy-2,3,5,6-tetrafluoro-4′-methylbiphenyl (10d): ^{1}H NMR ($CDCl_3$): δ 7.31 (d, 2H, $^3J_{HH}$ 8.1 Hz, C_{Ar}–*H*), 7.28 (d, 2H, $^3J_{HH}$ 8.3 Hz, C_{Ar}–*H*), 4.09 (s, 3H, CH_3O), 2.40 (s, 3H, CH_3Ar). ^{19}F NMR ($CDCl_3$): δ –159.5 (dd, 2F, $^3J_{FF}$ 22.2 Hz, $^5J_{FF}$ 8.8 Hz, F-2,6), –146.4 (dd, 2F, $^3J_{FF}$ 22.0 Hz, $^5J_{FF}$ 8.6 Hz, F-3,5). ^{19}F NMR (toluene): δ –158.5 (dd, 2F, $^3J_{FF}$ 22.2 Hz, $^5J_{FF}$ 8.4 Hz, F-2,6), –145.5 (dd, 2F, $^3J_{FF}$ 22.2 Hz, $^5J_{FF}$ 8.4 Hz, F-3,5). HRMS calcd for $C_{14}H_{10}F_4O$: 270.0668, found: 270.0667.

1-Ethoxy-2,3,5,6-tetrafluoro-4′-methylbiphenyl (10e): ^{1}H NMR ($CDCl_3$): δ 7.32 (d, 2H, $^3J_{HH}$ 8.1 Hz, C_{Ar}–*H*), 7.27 (d, 2H, $^3J_{HH}$ 8.3 Hz, C_{Ar}–*H*), 4.32 (q, $^3J_{HH}$ 7.0 Hz, 2H, CH_2O), 2.40 (s, 3H, CH_3Ar), 1.44 (t, 3H, $^3J_{HH}$ 7.0 Hz, CH_3). ^{19}F NMR ($CDCl_3$): δ –158.8 (dd, 2F, $^3J_{FF}$ 22.2 Hz, $^5J_{FF}$ 8.8 Hz, F-2,6), –146.6 (dd, 2F, $^3J_{FF}$ 22.4 Hz, $^5J_{FF}$ 9.1 Hz, F-3,5). ^{19}F NMR (toluene): δ –157.8 (dd, 2F, $^3J_{FF}$ 22.2, $^5J_{FF}$ 8.0 Hz, F-2,6), –145.5 (dd, 2F, $^3J_{FF}$ 21.6 Hz, $^5J_{FF}$ 7.3 Hz, F-3,5). HRMS calcd for $C_{15}H_{12}F_4O$: 284.0824; found: 284.0822.

1-Propoxy-2,3,5,6-tetrafluoro-4′-methylbiphenyl (10f): ^{1}H NMR ($CDCl_3$): δ 7.32 (d, 2H, $^3J_{HH}$ 8.3 Hz, C_{Ar}–*H*), 7.27 (d, 2H, $^3J_{HH}$ 8.3 Hz, C_{Ar}–*H*), 4.21 (t, 2H, $^3J_{HH}$ 6.7 Hz, CH_2O), 2.40 (s, 3H, CH_3Ar), 1.82 (qt, 2H, $^3J_{HH}$ 7.4, $^3J_{HH}$ 6.7 Hz, CH_2), 1.06 (t, 3H, $^3J_{HH}$ 7.4 Hz, CH_3). ^{19}F NMR ($CDCl_3$): δ –158.8 (dd, 2F, $^3J_{FF}$ 22.2 Hz, $^5J_{FF}$ 8.8 Hz, F-2,6), –146.6 (dd, 2F, $^3J_{FF}$ 22.2 Hz, $^5J_{FF}$ 8.8 Hz, F-3,5). ^{19}F NMR (toluene): δ –157.8 (dd, 2F, $^3J_{FF}$ 22.2 Hz, $^5J_{FF}$ 8.4 Hz, F-2,6), –145.5 (dd, 2F, $^3J_{FF}$ 22.4 Hz, $^5J_{FF}$ 8.2 Hz, F-3,5). HRMS calcd for $C_{16}H_{14}F_4O$: 298.0981; found: 298.0980.

1-Isoropoxy-2,3,5,6-tetrafluoro-4′-methylbiphenyl (10j): ^{1}H NMR ($CDCl_3$): δ 7.32 (d, 2H, $^3J_{HH}$ 8.4 Hz, C_{Ar}–*H*), 7.27 (d, 2H, $^3J_{HH}$ 8.1 Hz, C_{Ar}–*H*), 4.57 (septet, 1H, $^3J_{HH}$ 6.1 Hz, C*H*), 2.40 (s, 3H, CH_3Ar), 1.40 (d, 6H, $^3J_{HH}$ 6.1 Hz, CH_3). ^{19}F NMR ($CDCl_3$): δ –157.8 (dd, 2F, $^3J_{FF}$ 22.4 Hz, $^5J_{FF}$ 9.1 Hz, F-2,6), –146.7 (dd, 2F, $^3J_{FF}$ 22.4 Hz, $^5J_{FF}$ 9.1 Hz, F-3,5). ^{19}F NMR (toluene): δ –156.7 (dd, 2F, $^3J_{FF}$ 22.6 Hz, $^5J_{FF}$ 8.8 Hz, F-2,6), –145.5 (dd, 2F, $^3J_{FF}$ 22.6 Hz, $^5J_{FF}$ 8.8 Hz, F-3,5). HRMS calcd for $C_{16}H_{14}F_4O$: 298.0981; found: 298.0982.

1-Butoxy-2,3,5,6-tetrafluoro-4′-methylbiphenyl (10i): ^{1}H NMR ($CDCl_3$): δ 7.35 (d, 2H, $^3J_{HH}$ 8.4 Hz, C_{Ar}–*H*), 7.30 (d, 2H, $^3J_{HH}$ 8.1 Hz, C_{Ar}–*H*), 4.28 (t, 2H, $^3J_{HH}$ 6.5 Hz), 2.43 (s, 3H, CH_3Ar), 1.81 (tt, 2H, $^3J_{HH}$ 7.6 Hz, $^3J_{HH}$ 6.5 Hz,), 1.55 (tq, 2H, $^3J_{HH}$ 7.6 Hz, $^3J_{HH}$ 7.4 Hz), 1.00 (t, 3H, $^3J_{HH}$ 7.4 Hz). ^{19}F ($CDCl_3$): δ –158.8 (dd, 2F, $^3J_{FF}$ 22.2 Hz, $^5J_{FF}$ 8.8 Hz, F-2,6), –146.6 (dd, 2F,

$^3J_{FF}$ 22.2 Hz, $^5J_{FF}$ 8.8 Hz, F-3,5). ^{19}F NMR (toluene): δ –157.8 (dd, 2F, $^3J_{FF}$ 22.4 Hz, $^5J_{FF}$ 9.1 Hz, F-2,6), –145.5 (dd, 2F, $^3J_{FF}$ 22.6 Hz, $^5J_{FF}$ 8.8 Hz, F-3,5). HRMS calcd for $C_{17}H_{16}F_4O$: 312.1137; found: 312.1138.

1-*tert*-Butoxy-2,3,5,6-tetrafluoro-4′-methylbiphenyl (10k): ^{1}H NMR ($CDCl_3$): δ 7.32 (d, 2H, $^3J_{HH}$ 8.0 Hz, C_{Ar}–*H*), 7.28 (d, 2H, $^3J_{HH}$ 8.0 Hz, C_{Ar}–*H*), 2.40 (s, 3H, CH_3Ar), 1.44 (s, 9H, $OC(CH_3)_3$). ^{19}F NMR ($CDCl_3$): δ –146.9 (dd, 2F, $^3J_{FF}$ 23.1 Hz, $^5J_{FF}$ 9.3 Hz, F-3,5), –153.2 (dd, 2F, $^3J_{FF}$ 23.1 Hz, $^5J_{FF}$ 8.8 Hz, F-2,6). ^{19}F NMR (toluene): δ –145.6 (dd, 2F, $^3J_{FF}$ 23.5 Hz, $^5J_{FF}$ 9.3 Hz, F-3,5), –152.2 (dd, 2F, $^3J_{FF}$ 23.3 Hz, $^5J_{FF}$ 9.1 Hz, F-2,6). HRMS calcd for $C_{16}H_{13}F_4O$: 297.0896 (m/z –CH_3); found: 297.0897.

1-Benzyloxy-2,3,5,6-tetrafluoro-4′-methylbiphenyl (10h): ^{1}H NMR ($CDCl_3$): δ 7.46 (s, 1H, H^{para}), 7.41 (d, 2H, $^3J_{HH}$ 5.5 Hz, H^{ortho}), 7.37 (d, 2H, $^3J_{HH}$ 5.5 Hz, H^{meta}), 7.31 (d, 2H, $^3J_{HH}$ 7.3 Hz, C_{Ar}–*H*), 7.27 (d, 2H, $^3J_{HH}$ 8.3 Hz, C_{Ar}–*H*), 5.28 (s, 2H, CH_2O), 2.42 (s, 3H, CH_3Ar). ^{19}F NMR ($CDCl_3$): δ –157.8 (dd, 2F, $^3J_{FF}$ 22.4 Hz, $^5J_{FF}$ 9.1 Hz, F-2,6), –146.3 (dd, 2F, $^3J_{FF}$ 22.4 Hz, $^5J_{FF}$ 8.6 Hz, F-3,5). ^{19}F NMR (toluene): δ –156.8 (dd, 2F, $^3J_{FF}$ 22.6 Hz, $^5J_{FF}$ 8.4 Hz, F-2,6), –145.4 (dd, 2F, $^3J_{FF}$ 22.6 Hz, $^5J_{FF}$ 8.4 Hz, F-3,5). HRMS calcd for $C_{20}H_{14}F_4O$: 346.0981; found: 346.0982.

1-(2-Phenylethyloxy)-2,3,5,6-tetrafluoro-4′-methylbiphenyl (10l): ^{1}H NMR ($CDCl_3$): δ 7.40–7.24 (m, 5H, C_{Ar1}–*H*), 7.34 (m, 2H, C_{Ar}–*H*), 7.32 (m, 2H, C_{Ar}–*H*), 4.50 (t, 2H, $^3J_{HH}$ 7.1 Hz, CH_2), 3.17 (t, 2H, $^3J_{HH}$ 7.1 Hz, CH_2), 2.44 (s, 3H, CH_3Ar). ^{19}F NMR ($CDCl_3$): δ –158.6 (dd, 2F, $^3J_{FF}$ 22.4 Hz, $^5J_{FF}$ 8.6 Hz, F-2,6), –146.5 (dd, 2F, $^3J_{FF}$ 22.4 Hz, $^5J_{FF}$ 8.6 Hz, F-3,5). ^{19}F NMR (toluene): δ –157.6 (dd, 2F, $^3J_{FF}$ 22.4 Hz, $^5J_{FF}$ 8.6 Hz, F-2,6), –145.5 (dd, 2F, $^3J_{FF}$ 22.4 Hz, $^5J_{FF}$ 8.6 Hz, F-3,5). HRMS calcd for $C_{21}H_{16}F_4O$: 360.1137; found: 360.1137.

1-Allyloxy-2,3,5,6-tetrafluoro-4′-methylbiphenyl (10c): ^{1}H NMR ($CDCl_3$): δ 7.34 (d, 2H, $^3J_{HH}$ 8.5 Hz, C_{Ar}–*H*), 7.29 (d, 2H, $^3J_{HH}$ 8.2 Hz, C_{Ar}–*H*), 6.08 (ddt, 1H, $^3J_{HH}$ 6.1 Hz, $^3J_{HH}$ 10.4 Hz, $^3J_{HH}$ 17.1 Hz, H), 5.43 (ddd, 1H, $^3J_{HH}$ 17.2 Hz, $^4J_{HH}$ 2.8 Hz, $^2J_{HH}$ 1.4 Hz, $H^{2\text{-}E}$), 5.34 (ddd, 1H, $^3J_{HH}$ 10.4 Hz, $^4J_{HH}$ 2.3 Hz, $^2J_{HH}$ 1.1 Hz, $H^{2\text{-}Z}$), 4.76 (d, 2H, $^3J_{HH}$ 6.0 Hz, CH_2O), 2.40 (s, 3H, CH_3Ar). ^{19}F NMR ($CDCl_3$): δ –158.1 (dd, 2F, $^3J_{FF}$ 22.4 Hz, $^5J_{FF}$ 8.6 Hz, F-2,6), –146.5 (dd, 2F, $^3J_{FF}$ 22.4 Hz, $^5J_{FF}$ 9.1 Hz, F-3,5). ^{19}F NMR (toluene): δ –157.1 (dd, 2F, $^3J_{FF}$ 22.6 Hz, $^5J_{FF}$ 8.0 Hz, F-2,6), –145.5 (dd, 2F, $^3J_{FF}$ 22.6 Hz, $^5J_{FF}$ 8.0 Hz, F-3,5). HRMS calcd for $C_{16}H_{12}F_4O$: 296.0824; found: 296.0823.

1-Phenoxy-2,3,5,6-tetrafluoro-4′-methylbiphenyl (10m): ^{1}H NMR ($CDCl_3$): δ 7.36 (d, 2H,

$^3J_{HH}$ 7.3 Hz, C_{Ar1}–*H*), 7.30 (d, 2H, $^3J_{HH}$ 8.3 Hz, C_{Ar}–*H*), 7.48 (s, 2H, H^{meta}), 7.02 (d, 2H, $^3J_{HH}$ 8.1 Hz, H^{ortho}), 7.12 (t, 1H, $^3J_{HH}$ 7.3 Hz, H^{para}), 2.42 (s, 3H, CH_3Ar). ^{19}F NMR ($CDCl_3$): δ –155.9 (dd, 2F, $^3J_{FF}$ 22.4 Hz, $^5J_{FF}$ 9.5 Hz, F-2,6), –145.1 (dd, 2F, $^3J_{FF}$ 22.4 Hz, $^5J_{FF}$ 9.5 Hz, F-3,5). ^{19}F NMR (toluene): δ –154.9 (dd, 2F, $^3J_{FF}$ 22.4 Hz, $^5J_{FF}$ 9.5 Hz, F-2,6), –144.1 (dd, 2F, $^3J_{FF}$ 22.9 Hz, $^5J_{FF}$ 9.5 Hz, F-3,5). HRMS calcd for $C_{19}H_{12}F_4O$: 332.0824; found: 332.0823.

1-(Pyrrol-1-yl)-2,3,5,6-tetrafluoro-4′-methylbiphenyl (10o): ^{1}H NMR ($CDCl_3$): δ 7.38 (d, 2H, $^3J_{HH}$ 8.3 Hz, C_{Ar}–*H*), 7.31 (d, 2H, $^3J_{HH}$ 8.8 Hz, C_{Ar}–*H*), 6.98 (ddd, 2H, $^3J_{HH}$ 4.2 Hz, $^4J_{HH}$ 2.1 Hz, $^4J_{HH}$ 2.1 Hz, C*H*), 6.41 (ddd, 2H, $^3J_{HH}$ 4.2 Hz, $^3J_{HH}$ 2.1 Hz, $^4J_{HH}$ 2.1 Hz, C*H*), 2.43 (s, 3H, CH_3Ar). ^{19}F NMR ($CDCl_3$): δ –151.5 (dd, 2F, $^3J_{FF}$ 22.4 Hz, $^5J_{FF}$ 9.9 Hz, F-2,6), –144.4 (dd, 2F, $^3J_{FF}$ 22.9 Hz, $^5J_{FF}$ 9.9 Hz, F-3,5). ^{19}F NMR (toluene): δ –150.8 (dd, 2F, $^3J_{FF}$ 22.6 Hz, $^5J_{FF}$ 9.7 Hz, F-2,6), –143.9 (dd, 2F, $^3J_{FF}$ 22.4 Hz, $^5J_{FF}$ 9.5 Hz, F-3,5). HRMS calcd for $C_{17}H_{11}F_4N$: 305.0828; found: 305.0829.

1-(Pyrazol-1-yl)-2,3,5,6-tetrafluoro-4′-methylbiphenyl (10p): ^{1}H NMR ($CDCl_3$): δ 7.87 (d, 1H, $^3J_{HH}$ 1.7 Hz, H^3), 7.77 (ddd, 2H, $^3J_{HH}$ 2.4 Hz, $^4J_{HH}$ 1.4 Hz, $^4J_{HH}$ 1.1 Hz, H^5), 7.40 (d, 2H, $^3J_{HH}$ 8.3 Hz, C_{Ar}–*H*), 7.34 (d, 2H, $^3J_{HH}$ 8.1 Hz, C_{Ar}–*H*), 6.57 (dd, 1H, $^3J_{HH}$ 2.5 Hz, $^3J_{HH}$ 1.9 Hz, H^4), 2.44 (s, 3H, CH_3Ar). ^{19}F NMR ($CDCl_3$): δ –150.2 (dd, 2F, $^3J_{FF}$ 20.7 Hz, $^5J_{FF}$ 8.6 Hz, F-2,6), –144.1 (dd, 2F, $^3J_{FF}$ 20.9 Hz, $^5J_{FF}$ 8.9 Hz, F-3,5). ^{19}F NMR (toluene): δ –149.2 (dd, 2F, $^3J_{FF}$ 21.6 Hz, $^5J_{FF}$ 9.5 Hz, F-2,6), –143.8 (dd, 2F, $^3J_{FF}$ 21.1 Hz, $^5J_{FF}$ 9.1 Hz, F-3,5). HRMS calcd for $C_{16}H_{10}F_4N_2$: 306.0780; found: 306.0781.

1-(Indol-1-yl)-2,3,5,6-tetrafluoro-4′-methylbiphenyl (10n): ^{1}H NMR ($CDCl_3$): δ 7.67 (dd, 1H, $^3J_{HH}$ 8.3 Hz, $^4J_{HH}$ 1.2 Hz, H^4), 7.40 (d, 2H, $^3J_{HH}$ 8.3 Hz, C_{Ar}–*H*), 7.30 (d, 2H, $^3J_{HH}$ 8.0 Hz, C_{Ar}–*H*), 7.23 (d, 1H, $^3J_{HH}$ 1.5 Hz, H^2), 7.22–7.16 (m, 3H, $H^{5,6,7}$), 6.76 (dd, 1H, $^3J_{HH}$ 3.4 Hz, $^4J_{HH}$ 0.6 Hz, H^4), 2.41 (s, 3H, CH_3Ar). ^{19}F NMR ($CDCl_3$): δ –147.8 (dd, 2F, $^3J_{FF}$ 21.1 Hz, $^5J_{FF}$ 9.1 Hz, F-2,6), –144.1 (dd, 2F, $^3J_{FF}$ 20.7 Hz, $^5J_{FF}$ 9.1 Hz, F-3,5). ^{19}F NMR (toluene): δ –146.8 (dd, 2F, $^3J_{FF}$ 22.4 Hz, $^5J_{FF}$ 9.9 Hz, F-2,6), –143.3 (dd, 2F, $^3J_{FF}$ 22.0 Hz, $^5J_{FF}$ 9.5 Hz, F-3,5). HRMS calcd for $C_{21}H_{13}F_4N$: 355.0984; found: 355.0985.

7. Cross-coupling of K[4-$RC_6F_4BF_3$] (1b-p) and K[$C_6F_5BF_3$](1a) (1:1) with 11.

A glass vessel equipped with a magnetic stir bar was flushed with dry argon, and charged with $Pd(OAc)_2$ (1.9 mg, 0.008 mmol), P(*t*-Bu)$_3$ (3.4 mg, 0.017 mmol), a solution of **11** (142.8 mg, 0.835 mmol in toluene (143 mg)), Ag_2O (92.8 mg, 0.400 mmol), K[4-$RC_6F_4BF_3$] (**1b-p**, 0.200 mmol), K[$C_6F_5BF_3$] (**1a**, 54.8 mg, 0.200 mmol, K_2CO_3 (92.4 mg, 0.668 mmol) and toluene (1 mL). The vessel was sealed with AluCap©, the resulted suspension was stirred for 5-15 min at

100 °C (bath) and cooled to 25 °C. After injection of $C_6H_5CF_3$ (10.0 µL, 0.082 mmol) (internal reference) products were analyzed by ^{19}F NMR spectroscopy (Table 3).

8. Cross-coupling of 1a with 11 in the presence of Ag*n*Y.

A glass vessel equipped with a magnetic stir bar was flushed with dry argon, and charged with $Pd(OAc)_2$ (1.9 mg, 0.008 mmol), P(*t*-Bu)$_3$ (3.4 mg, 0.017 mmol), a solution of **11** (28.5 mg, 0.167 mmol in toluene (29 mg)), Ag$_n$Y (0.200 mmol), **1a** (54.8 mg, 0.200 mmol, K_2CO_3 (46.2 mg, 0.334 mmol) and toluene (1 mL). The vessel was sealed with AluCap©, the suspension was stirred for 1 h at 100 °C (bath) and cooled to 25 °C. After injection of $C_6H_5CF_3$ (10.0 µL, 0.082 mmol) (internal reference) the mother liquor was analyzed by ^{19}F NMR spectroscopy (Table 4).

9. Attempted reaction of 1a with K_2CO_3 and Ag_2O in toluene.

A glass vessel equipped with a magnetic stir bar was flushed with dry argon, charged with **1a** (54.8 mg, 0.20 mmol), K_2CO_3 (45.5 mg, 0.33 mmol), Ag_2O (46.2 mg, 0.20 mmol), toluene (1 mL) and sealed with AluCap©. The suspension was stirred at 100 °C (bath) for 10 min. After cooling, $C_6H_5CF_3$ (10.0 µL, 0.082 mmol) (internal reference) was added. The suspension was filtered, the precipitate was washed with toluene (1.5 mL) and CCl_4 (2 mL), dried in air and extracted with acetone (2 mL). Extract contained only **1a** (0.20 mmol) (^{19}F NMR).

10. References.

1. Santiago Cintrón, M.; Green, O.; Burstyn, J. N. *Inorg. Chem.* **2012,** *51*, 2737-2746.
2. Frohn, H. J.; Franke, H.; Fritzen, P.; Bardin, V. V. *J. Organomet. Chem.* **2000,** *598*, 127-135.
3. Adonin, N. Y.; Babushkin, D. E.; Parmon, V. N.; Bardin, V. V.; Kostin, G. A.; Mashukov, V. I.; Frohn, H.-J. *Tetrahedron* **2008,** *64*, 5920-5924.
4. Shabalin, A. Y.; Adonin, N. Y.; Bardin, V. V.; Prikhod'ko, S. A.; Timofeeva, M. N.; Bykova, M. V.; Parmon, V. N. *J. Fluorine Chem.* **2013,** *149*, 82-87.
5. Shabalin, A. Y.; Adonin, N. Y.; Bardin, V. V.; Taran, O. P.; Ayusheev, A. B.; Parmon, V. N. *J. Fluorine Chem.* **2013,** *156*, 290-297.
6. Abo-Amer, A.; Adonin, N. Y.; Bardin, V. V.; Fritzen, P.; Frohn, H.-J.; Steinberg, C. *J. Fluorine Chem.* **2004,** *125*, 1771-1778.
7. Shabalin, A. Y.; Adonin, N. Y.; Bardin, V. V.; Parmon, V. N. *Tetrahedron* **2014,** *70*, 3720-3725.
8. Ohashi, M.; Doi, R.; Ogoshi , S. *Chem. Eur. J.* **2014,** *20*, 2040-2048.
9. Lesieur, M.; Lazreg, F.; Cazin, C. S. J. *Chem. Commun.* **2014,** *50*, 8927-8929.
10. Frohn, H.-J.; Adonin, N. Y.; Bardin, V. V.; Starichenko, V. F. *J. Fluorine Chem.* **2002,** *117*, 115-120.

11. Miao, T.; Wang, L. *Adv. Synth. Catal.* **2014,** *356*, 429-436.
12. Berger, S.; Braun, S.; Kalinowski, H.-O. *NMR-Spektroskopie von Nichtmetallen. Bd 4.* ^{19}*F-NMR-Spektroskopie;* Thieme Verlag: Stuttgart, Germany, 1994.
13. Gordon Fearon, F. W.; Gilman, H. *J. Organomet. Chem.* **1967,** *10*, 535-537.
14. Zhu, X.; Li, F.; Su, W. *Tetrahedron Lett.* **2013,** *54*, 1285-1289.
15. James, J. H.; Peach, M. E.; Williams, C. R. *J. Fluorine Chem.* **1985,** *27*, 91-104.
16. Chambers, R. D.; Spring, D. J. *Tetrahedron* **1971,** *27*, 669-680.
17. Artamkina, G. A.; Sazonov, P. K.; Ivushkin, V. A.; Beletskaya, I. P. *Chem. Eur. J.* **1998,** *4*, 1169-1178.
18. Bruce, D. W.; Metrangolo, P.; Meyer, F.; Pilati, T.; Präsang, C.; Resnati, G.; Terraneo, G.; Wainwright, S. G.; Whitwood, A. C. *Chem. Eur. J.* **2010,** *16*, 9511-9524.
19. Bardin, V. V.; Rogoza, L. N.; Stennikova, I. V.; Furin, G. G. *J. Fluorine Chem.* **1992,** *59*, 165-177.
20. Budnik, A. G.; Senchenko, T. V.; Shteingarts, V. D. *Zh. Org. Khim.* **1974,** *10*, 344-350.
21. Liu, C.; Cao, L.; Yin, X.; Xu, H.; Zhang, B. *J. Fluorine Chem.* **2013,** *156*, 51-60.
22. Cano, R.; Ramón, D. J.; Yus, M. *J. Org. Chem.* **2010,** *76*, 654-660.
23. Fujii, S.; Maki, Y.; Kimoto, H. *J. Fluorine Chem.* **1989,** *43*, 131-144.
24. Liu, C.; Wang, H.; Xing, X.; Xu, Y.; Ma, J.-A.; Zhang, B. *Tetrahedron Lett.* **2013,** *54*, 4649-4652.
25. Adonin, N. Y.; Shabalin, A. Y.; Bardin, V. V. *J. Fluorine Chem.* **2014,** *168*, 111-120.
